# A Survey of Transposon Landscapes in the Putative Ancient Asexual Ostracod *Darwinula stevensoni*

**DOI:** 10.3390/genes12030401

**Published:** 2021-03-11

**Authors:** Isa Schön, Fernando Rodriguez, Matthew Dunn, Koen Martens, Michael Shribak, Irina R. Arkhipova

**Affiliations:** 1 Freshwater Biology, OD Nature, Royal Belgian Institute of Natural Sciences, Vautierstraat 29, B-1000 Brussels, Belgium; darwinula@gmail.com; 2 Center of Environmental Sciences (CMK), University of Hasselt, 3590 Diepenbeek, Belgium; 3 Marine Biological Laboratory, 7 MBL Street, Woods Hole, MA 02543, USA; frodriguez@mbl.edu (F.R.); mjdunn@umass.edu (M.D.); mshribak@mbl.edu (M.S.); iarkhipova@mbl.edu (I.R.A.); 4 Dept Biology, University of Ghent, 9000 Ghent, Belgium

**Keywords:** transposable elements, retrotransposons, DNA transposons, crustaceans, asexuality

## Abstract

How asexual reproduction shapes transposable element (TE) content and diversity in eukaryotic genomes remains debated. We performed an initial survey of TE load and diversity in the putative ancient asexual ostracod *Darwinula stevensoni*. We examined long contiguous stretches of DNA in clones from a genomic fosmid library, totaling about 2.5 Mb, and supplemented these data with results on TE abundance and diversity from an Illumina draft genome. In contrast to other TE studies in putatively ancient asexuals, which revealed relatively low TE content, we found that at least 19% of the fosmid dataset and 26% of the genome assembly corresponded to known transposons. We observed a high diversity of transposon families, including LINE, gypsy, PLE, mariner/Tc, hAT, CMC, Sola2, Ginger, Merlin, Harbinger, MITEs and helitrons, with the prevalence of DNA transposons. The predominantly low levels of sequence diversity indicate that many TEs are or have recently been active. In the fosmid data, no correlation was found between telomeric repeats and non-LTR retrotransposons, which are present near telomeres in other taxa. Most TEs in the fosmid data were located outside of introns and almost none were found in exons. We also report an N-terminal Myb/SANT-like DNA-binding domain in site-specific R4/Dong non-LTR retrotransposons. Although initial results on transposable loads need to be verified with high quality draft genomes, this study provides important first insights into TE dynamics in putative ancient asexual ostracods.

## 1. Introduction

The influence of reproductive mode on transposon content and distribution in eukaryotic genomes remains a subject of debate as it seems to be shaped by several evolutionary forces acting in opposite directions. Sexual reproduction is expected to greatly facilitate the spread of vertically transmitted transposons in populations [1], as has been experimentally shown in yeast (see for example [2] or [3]).

All asexuals originate from sexual ancestors. If they inherited transposons, the absence of meiotic recombination should lead to reduced efficacy of selection and accumulation of deleterious mutations [4] and transposons, at least in finite populations [5,6]. Empirical evidence for these predictions comes from non-recombining sex chromosomes (e.g., [7]) and other non-recombining parts of the genome [8,9]. Such unrestrained transposable element (TE) proliferation should eventually drive asexual lineages to extinction unless asexual hosts can keep TE copy numbers under tight control [10,11,12], with putative ancient asexual bdelloids as the most striking example [10,13,14] of having reduced numbers of vertically transmitted retrotransposons while they still harbor some DNA transposons that can be horizontally transmitted. In line with these predictions, sexual *Daphnia pulex* have higher loads of DNA transposons [15], LTR retrotransposons [16] or more insertion polymorphisms of transposons [17] than their asexual counterparts. Comparative genomic studies on asexual and sexual arthropods [18], nematodes [19], evening primroses [20] and green algae [21], however, did not show any significant effect of the reproductive mode on transposon content and evolution. Other genomic studies reported higher transposon loads in asexuals, as for example for root-knot nematodes [22] or parasitic wasps [23].

These contrasting results can be partly explained by different lineage ages [24]. Purging of transposons in asexuals can take a very long time, and transposons are expected to accumulate if they cannot be removed [25]. Theoretical studies [26,27] and an ever-increasing number of genomic studies from different host organisms [28,29] suggest that additional factors besides the reproductive mode will also influence transposon diversity and load, such as initial transposon load in sexual ancestors, DNA methylation, population size [30], environmental fluctuations [27], strengths of selection and drift [19], and molecular defense mechanisms against transposable elements. DNA transposons are more frequently transmitted horizontally (as in the example of insects; [31]) than retrotransposons, and are expected to be less affected by the reproductive mode.

To better understand transposon evolution, their molecular characteristics and biological effects, additional in vivo and in vitro studies are thus required [28], especially from non-model organisms. The continuous increase of genomic data has also revealed the extent of lineage-specific transposon diversities, which further increases the methodological challenges of analyzing these elements [28] and provides additional motivation for studying them in a wide range of organisms. 

**Figure 1:**
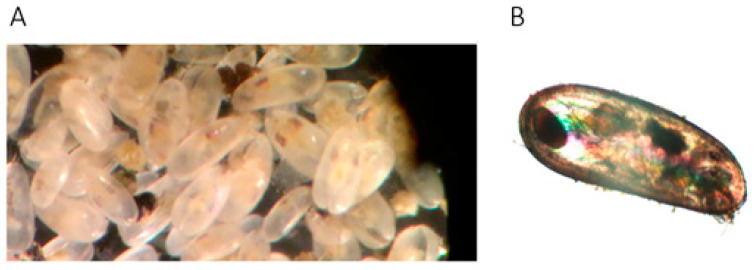
*Darwinula stevensoni*. (**A**) A sample of multiple individuals of *Darwinula stevensoni*. Taken by Jeroen Vendericks. (**B**) Lateral view of the carapace of an individual *D*. *stevensoni*. This picture was taken with the polychromatic polarization microscope [40] with a 4× objective lens and a DP73 camera. The total length of the animals is around 800 μm. In the left corner, an embryo in the brooding pouch is visible.

The ostracod family Darwinulidae is one of the few examples of putative ancient asexual animals [32,33,34,35], to which bdelloid rotifers also belong [36]. Fossil data indicate that some darwinulids might have been asexual for 200 million years [37], and the type species of this family that is investigated here, *Darwinula stevensoni* (Figure 1), might have been asexual for about 25 million years [38]. There is only one study on transposable elements of darwinulid ostracods describing novel LINE-like retroelements [39], and long, contiguous genome assemblies from these ostracods are not yet available. 

Here, we used long DNA sequences of *D. stevensoni* based on selected clones of a genomic fosmid library, totaling around 2.5 Mb, for addressing four aims: (1) To gain initial insights into TE content, diversity and activity in darwinulid ostracod genomes and confirm these preliminary results with data from an Illumina draft genome of *D. stevensoni*; (2) to examine possible links of TEs with telomeres; (3) to compare the location of TEs to coding gene regions (CDS); (4) to assess the possible impact of anciently asexual reproduction on TE landscapes of non-marine ostracods. We observed a high diversity of TEs in the putative ancient asexual ostracod *D. stevensoni*, and a high prevalence of 19–26% in the surveyed fosmid and draft genome DNA sequence data. Most TEs were located outside of coding regions, had no link to telomeres and showed evidence of recent activity. Our results provide first indications that putative ancient asexual ostracods might not be able to efficiently purge TEs from their genomes.

## 2. Materials and Methods

### 2.1. Construction, Screening and Sequencing of a Genomic Fosmid Library

A genomic fosmid library of *D. stevensoni* was constructed at Clemson University (USA) from 1000 pooled individual ostracods, sampled from the monoclonal Belgian population in Hollandersgatkreek, because of the small size of these ostracods (L = ca. 0.8 mm, see Figure 1A,B). Ostracods were first incubated for 10 days in pure water to evacuate any possible contaminants from the gut. High molecular weight DNA was isolated, randomly fragmented, end-repaired, phosphorylated, and size selected by pulsed-field gel electrophoresis. Size-selected fragments were ligated with the linearized dephosphorylated fosmid vector pCC2FOS (NovoPro) and then packaged by lambda packing extracts and plated on T1-phage resistant *E. coli*. A total of 18,432 recombinant colonies were randomly picked with a Genetix Q-bot robot and stored as individual clones at −80 °C in 384-well microtiter plates. Fosmids have an average insert size of 35 to 45 kbp, as was confirmed by randomly sampling 48 clones. Preliminary evaluation of *D. stevensoni* from the same Belgian population with flow cytometry and DAPI staining revealed a genome size of 0.86 to 0.93 pg, equaling 840 to 900 Million bp (Paczesniak et al., unpublished data). Karyological studies have shown that *D. stevensoni* nuclei contain 22 dot-like chromosomes [41] that cannot be visually grouped into homologous pairs, making it impossible to infer the ploidy level of *D. stevensoni* from cytological observations.

To identify fosmids containing either TEs or telomeres, specific overgo probes for LINE-like and mariner-like elements and telomeric repeats were developed (see Table S1 for details) from published data on two LINE-like elements of *D. stevensoni* [39] and unpublished data on TEs from non-marine ostracods, which had been acquired with PCR walking and Sanger sequencing using the general primers of [10]. Probes for telomeres were based on the universal arthropod telomeric repeat (TR) with the pentameric unit TTAGG [42]. We also screened for fosmids containing single-copy nuclear genes of *D. stevensoni* [43].

High density colony filters from the entire fosmid library were produced using a Genetix Q-bot in a 4 × 4 double-spotted array on GE HealthCare Hybond N+ membranes. Labeled PCR probes were used for hybridization of high-density colony filters and hits were called with Hybdecon v.01. The identity of fosmids having hits for TEs, single-copy nuclear genes and/or TRs was assessed. The hybridization experiments with the fosmid library revealed 86 fosmids with positive hits for the mariner probe, 18 for LINE-like *Daphne*, 33 for LINE-like *Syrinx* elements and 40 fosmids with hits for various nuclear genes (Table S2). A set of 96 fosmids, of which 13 contained positive hits for chosen TEs (five Daphne, five Syrinx, three mariner) and the other 83 for nuclear genes, were selected for further detailed analyses. DNA was extracted and prepared for high-throughput sequencing with the Ion XpressTM plus gDNA Fragment Library preparation kit and the Ion OneTouch™ 200 Template Kit v2, both from Life Technologies (Gent, Belgium). Individual fosmids were sheared, ligated to adapters with barcodes, size selected, pooled and used for an emulsion PCR. Fosmids were sequenced with the Ion PGM™ Sequencing 300 Kit on the Ion Torrent PGM using the Ion 316TM chip (Life Technologies). A total of 779 M DNA basepairs and 2,827,903 reads were generated, with a median read length of 290 bp and maximum length of 433 bp. Fosmids were demultiplexed, and quality filtering and assembly was conducted with CLC workbench (Qiagen; version 7.5.1) using default parameters. We selected 330 contigs with a minimum length of 2500 bp before vector removal resulting in a minimum length of 100 bp of these contigs for further analyses.

Another 11 fosmids were selected by hybridization with probes for single nuclear copy genes of potential horizontal origin (see Table S2) as part of the LATTECO project at the French Plant Genomic Resources Center (CNRGV; INRA facility, Toulouse, France). Sequence identities of the ends of positive fosmids were validated by PCR and Sanger sequencing. For PacBio RS II sequencing, 2 μg of each validated fosmid were tagged with PacBio tags and then pooled. The library was generated with the standard Pacific Biosciences library preparation protocol for 10 kb libraries and sequenced on one SMRT Cell using the P6v2 chemistry following the standard operating procedures of the manufacturer at the NGI (https://ngisweden.scilifelab.se/, accessed on 12 August 2020). Assembly of the PacBio RS II reads followed the HGAP workflow. The SMRT® Analysis (v2.2.0) software suite was used for HGAP implementation. Reads were first de-multiplexed and then aligned using BLASR against “*E. coli* str. K12 substr. DH10B, complete genome”. Identified *E. coli* reads and low-quality reads (read quality <0.80 and read length <500 bp) were removed. Filtered reads were then preassembled to generate long and highly accurate sequences. To perform this step, we separated the smallest and longest reads (e.g., >11 kbp) in order to correct read errors by mapping the smallest to the longest reads. Obtained sequences were filtered against vector sequences, and sequences were assembled into draft assemblies with the Celera assembler. As the final step of the HGAP workflow, “polishing” with Quiver significantly reduced remaining insertions and deletions and base substitution errors, resulting in high quality assemblies of a single contig per fosmid.

We also conducted BLAST searches [44] between all contigs to identify potentially overlapping redundant regions. The content of matching regions was further checked manually. If these included identical transposons, we considered them as evidence for recent transposition and kept the data. If (short) contigs fully matched other contigs, they were excluded. Likewise, short overlaps at the 5’ or 3’ end of fosmid sequences were also excluded if they showed more than 94% overlap with other contigs. In the absence of a phased reference genome, we can however not be certain if overlapping contigs originate from the same chromosome or from its homolog, as the allelic divergence is expected to be low, especially if the overlap is relatively short. To still account for possible bias from overlapping contig ends, we provide ranges for contigs and fosmid total lengths and estimates of exon, intron and TE content below. 

All fosmid sequence data have been submitted to NCBI (https://www.ncbi.nlm.nih.gov, accessed on 12 August 2020), accession numbers MW583466-MW583569.

### 2.2. Illumina Draft Genome of D. stevensoni

Besides the fosmid DNA sequence data, we also analyzed a recently published Illumina draft genome (European Nucleotide Archive accession number PRJEB38362) of *D. stevensoni* [45] for its TE content and diversity. This draft genome was assembled from DNA extractions of a single female, followed by whole genome amplification to provide sufficient material for the preparation of three 2 × 125 bp paired-end libraries (average insert sizes of 250–300, 550 and 700 bp), and two mate-pair libraries (average insert sizes of 3000 and 5000 bp), which were sequenced on an Illumina HiSeq 3000 system. The assembly has a size of 382.1 Mb, an N50 of 56.4 kb, an arthropod Benchmarking set of Universal Single-Copy Orthologs (BUSCO) score of 93.7% (complete single-copy genes) and consists of 62,118 scaffolds [45]. 

### 2.3. De Novo Identificiation of Ostracod TEs

We used the REPET package with default settings [46,47] for *de novo* TE identification and annotation of fosmid and draft genome data in three steps: detecting repeated sequences and potential TEs, clustering of these sequences, and generating consensus sequences for each cluster. Consensus sequences were classified following Wicker’s TE classification [48] and transposons were grouped by families. RepeatMasker [49] was applied for TE classification and plot building, using the local fosmid and genomic libraries of *D. stevensoni* from REPET. We constructed TE landscape divergence plots to evaluate the frequencies of different TE families in our dataset and estimate the Kimura substitution level of each TE family with adjusted CpG as a measure of TE activity over time. We also translated all fosmid DNA sequences and used the translated data in Censor (https://www.girinst.org/censor/, accessed on 12 August 2020) to reveal non-multicopy TEs and to classify TEs at the amino acid level. 

### 2.4. Assessing Insertion Sites of TEs from Fosmid Data

For obtaining preliminary information on the genomic location of TEs, we compared hybridization signals between fosmids with positive hits for telomeres and TEs. To assess if TEs were found in coding or non-coding genomic regions, we used our custom TE library with RepeatMasker to identify and soft-mask all TEs. The masked DNA sequence data were then used for gene predictions with Augustus [50] with *Drosophila melanogaster* as the species parameter. In regions identified as coding regions in the sequenced fosmids by Augustus [50], the lengths of exons and introns were calculated from the exact locations with LibreOffice Calc 6.4 to estimate the overall frequency of exons and introns in the DNA sequence data. To identify possible overlap of TEs and exons, we used BEDTools v2.29.2 [51] to compare the exact exon and intron locations in each fosmid with partial and complete TE locations as identified by Censor at the amino acid level. 

We calculated the frequency of introns and exons and TEs and overlap with exons and introns, respectively, per fosmid and visualized these as boxplots in ggplot2 [52] in R [53]. For selected fosmids, we used the output files of gene predictions with Augustus and TE identification by Censor to draw the positions of transcripts, exons, introns and TEs with Circos [54]. 

### 2.5. Estimating Single-Copy Gene Content in Fosmid Sequence Data

To assess the representativity of selected fosmids with regard to coding sequences, we conducted Benchmarking set of Universal Single-Copy Orthologs (BUSCO) v3.0.2 analyses [55] of all fosmid sequence data using Arthropoda_odb9 as a reference database for single-copy ortholog genes [56]. 

### 2.6. Search for Remote Homologies

To investigate the N-terminal domain in R4/Dong elements, we assembled a dataset of 5’-complete ORFs that included phylogenetically diverse additional sequences from GenBank identified by BLAST (accession numbers shown in Figure 6), aligned with MUSCLE v.3.8.31 [57], extracted the N-terminal part upstream of the reverse transcriptase domain, and used the multiple sequence alignment as a query on the HHpred server [58] with default settings. The C-terminal extension of *DsGypsy1* had no detectable homologs and was used as a standalone query. The seed alignment for PF00249 (*Myb_DNA*-binding) was downloaded from PFAM (http://pfam.xfam.org/, accessed on 17 December 2020). Structure-based alignments obtained with HHpred were visualized with Jalview v.2.11.1.3 [59] using the Clustal color scheme. 

## 3. Results

### 3.1. TE Diversity, Substitution Levels and Abundance 

**Figure 2:**
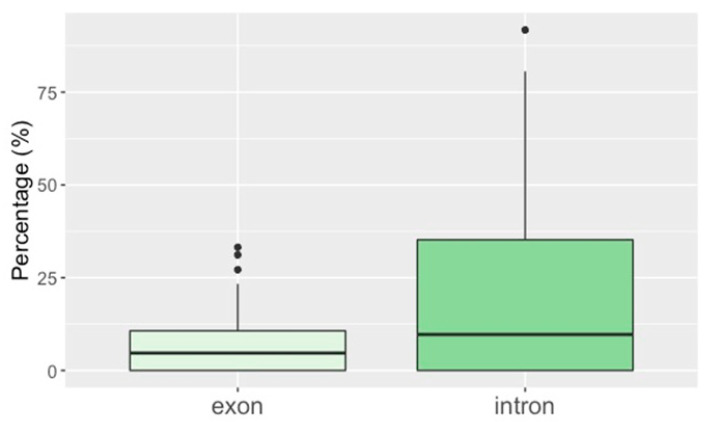
Box plots of intron and exon abundance. The frequency of introns and exons were calculated as % of total fosmid lengths. Boxes contain the interquartile range from the 25th to the 75th percentile, the horizontal line indicates the median, and vertical lines indicate minimum and maximum distributions of the data. Outliers are shown by dots.

The longest 341 contigs from 95 fosmids (Figure S1), providing a total of 2.39–2.55 Mbp with an average length of 7657–7472 bp and a median of 4242–4390 bp (Table S3A,B), taking into account possible overlaps, were further analyzed for TE and gene content (Figure 2). Details on potentially overlapping contigs are provided in Table S4.

**Figure 3:**
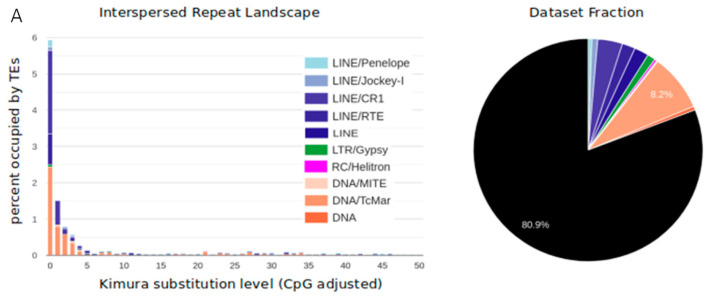

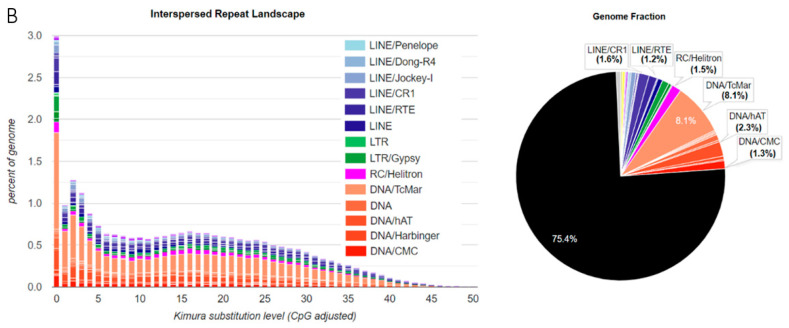
Landscape divergence plots (left hand side) and genome occupancy by known transposable elements (TEs) (right hand side) in *D. stevensoni***.** Divergences were calculated as Kimura substitution levels with adjusted CpG. Genome fraction of TEs was calculated after merging RepeatMasker and Censor outputs. (**A**, **top**) TEs in fosmid DNA sequence data. Genome fraction of TEs was calculated after merging RepeatMasker and Censor outputs. (**B**, **bottom**) TEs in the preliminary draft genome assembly [45]. The plot was constructed using the REPET library obtained with the Illumina assembly. The pie chart shows genome occupancy for TE categories occupying more than 1% of the genome. Concerning less frequent TEs in the draft genome, we found 0.2% of Penelope, 0.2% of Dong-R4 and 0.8% Jockey elements, 1.1% of gypsy and 0.6% of other LTR elements.

Our analyses revealed a high diversity of TEs in the selected clones from the fosmid library of *D. stevensoni* and the Illumina draft genome [45], including LINE-like retrotransposons, LTR retrotransposons, various cut-and-paste DNA transposons and Helitrons (Figure 3A,B). DNA transposons were best represented at 8.9% in the fosmids and 11.8% in the Illumina data, and belonged to Tc/mariner (most abundant, showing an excellent agreement at ~8% in both datasets; Figure 3A,B), hAT, Ginger, Merlin, Harbinger, and CMC-like DNA transposons, plus some uncharacterized DNA transposons. Similarly, for retrotransposons we found five major superfamilies of LINE-like elements (Jockey/I, CR1, L2, R4, RTE) with a total abundance of 4.2 and 2.8% in the fosmid and Illumina data, respectively, as well as gypsy-like (1% fosmids, 1.1% genome) and Penelope-like (0.3% fosmids, 0.2% genome) elements. Additionally, 4.2% of the fosmid sequence data (Table S5) were classified as non-LTR, whereas Helitrons constituted 0.3% of the fosmid and 1.5% of the genomic sequence data. The high diversity of TEs in the fosmid data is also illustrated in 38 selected contigs containing mariner-1 (Figure 5A) and mariner-2 DNA TEs (Figure 5C,D), a mixture of DNA, LINE-like and LTR TEs (Figure 5B), and LINE-like RTE (Figure 5E) and CR1 (Figure 5F) TEs.

**Figure 4:**
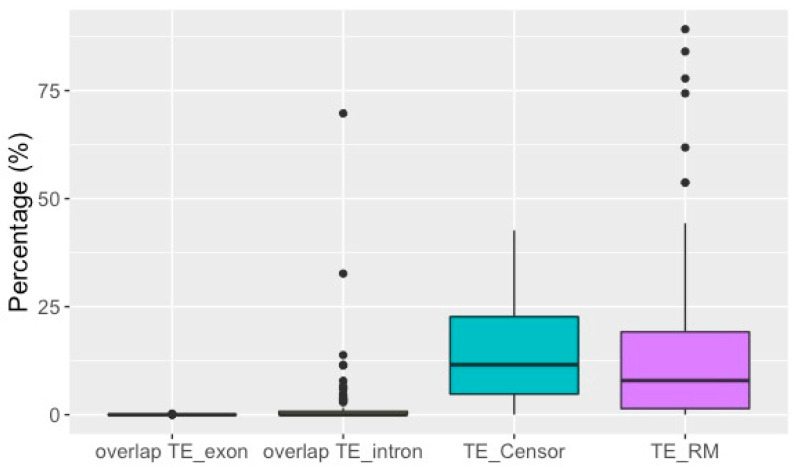
Box plots of TE abundance and overlap of TEs with exons and introns in fosmid data. TEs were identified with Censor from translated fosmid DNA sequences. All frequencies were calculated as % of total fosmid lengths. Boxes contain the interquartile range from the 25th to the 75th percentile, the horizontal line indicates the median, and vertical lines indicate minimum and maximum distributions of the data. Outliers are shown by dots.

The distribution of Kimura substitution levels on the TE divergence plot was negatively logarithmic in both datasets (Figure 3A,B), with most TEs having low substitution levels and only a few TEs showing high levels of substitution, indicating that most copies originated from relatively recent transposition events. In total, 19.1% of all fosmid DNA sequence data (Figure 3A) and 25.6% of the draft genome (Figure 3B) were comprised of known TE sequences. RepeatMasker and Censor estimated the median frequencies of TEs per fosmid as 8.6–9.4 and 11.7–12.8%, respectively, and average frequencies of 16.6–17.5 and 16.0–16.7%, respectively (Figure 4 and Table S3B). Average TE abundance estimated at the contig level was similar, with 13.7–14.7% (RepeatMasker) and 16.5–16.9% (Censor; Table S3A).

### 3.2. TE Insertion Sites in Fosmids and Their Relationship to Telomeres and Coding Regions

When comparing hybridization signals, there was no overlap between fosmids with a signal for telomeres and fosmids that contained TEs. With regard to coding regions, fosmids contained on average 6.4–6.7% exons and 20.1% introns, with a maximum of 91.2% introns (Figure 2). 

**Figure 5:**
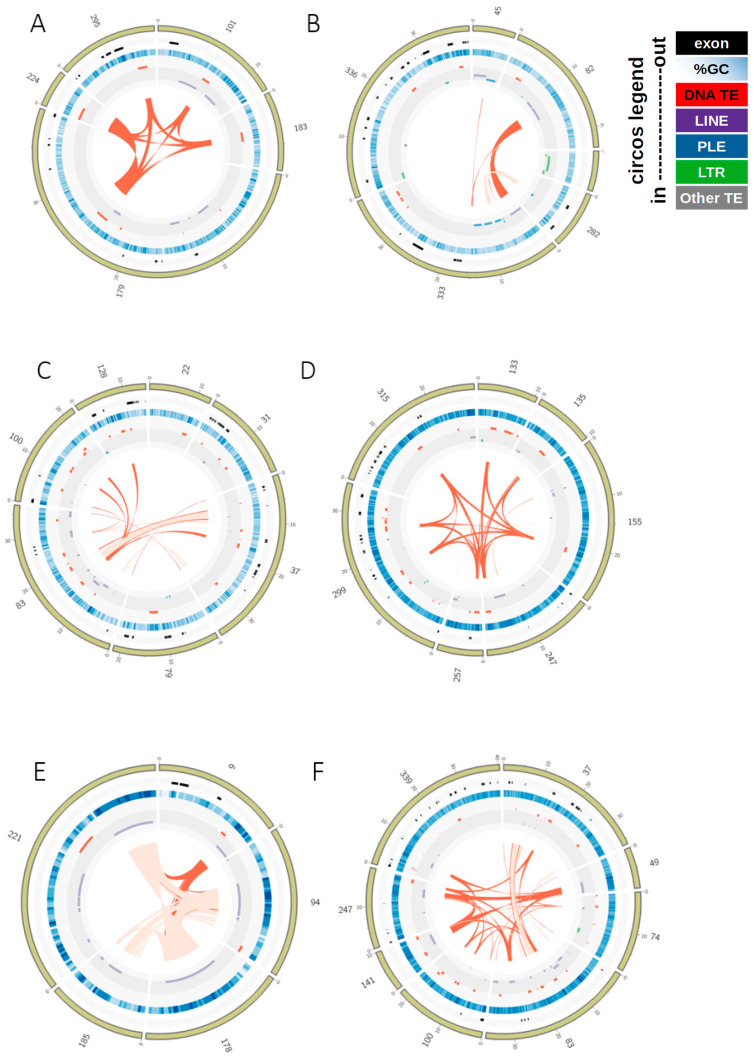
Sequence features of representative fosmid contigs. Features of contigs (marked outside in yellow) are plotted on tracks and their identities indicated as colored boxes shown in the figure legend. Shades of red inside the circles represent % identity from BLAST searches (dark color 100%, intermediate 90–99%, and light color 80–89%), indicating recent transposition (see also Table S4). Large numbers on the outside indicate contigs; smaller numbers, the position in kb. Translated TEs were identified with Censor; transcripts, exons and introns were predicted with Augustus; all features visualized with Circos. For further details on contigs, see Table S3A. (**A**) Contigs Ds_ctg224, Ds_ctg295, Ds_ctg101, Ds_ctg183 and Ds_ctg179 containing mariner-1 DNA TEs. (**B**) Contigs Ds_ctg 45, Ds_ctg 82, Ds_ctg 89, Ds_ctg 282, Ds_ctg 333 and Ds_ctg 336 with various TEs including DNA (mariner), LINE (PLE and CR1), and LTR (gypsy). (**C**,**D**) Contigs Ds_ctg22, Ds_ctg31, Ds_ctg37, Ds_ctg79, Ds_ctg83, Ds_ctg100 and Ds_ctg128 and Ds_ctg133, Ds_ctg135, Ds_ctg155, Ds_ctg247, Ds_ctg257, Ds_ctg299 and Ds_ctg315, respectively, displaying abundant mariner-2 TEs with high sequence similarities. (**E**,**F**) Contigs containing LINE-like TEs, including contigs Ds_ctg9, Ds_ctg94, Ds_ctg178, Ds_ctg185 and Ds_ctg221 containing RTE (**E**) and contigs Ds_ctg37, Ds_ctg49, Ds_ctg74, Ds_ctg83, Ds_ctg100, Ds_ctg141, Ds_ctg247 and Ds_ctg339 containing CR1 (**F**) TEs.

The second set of 11 fosmids that had been selected with probes for nuclear genes contained more exons and introns than the other fosmids (Figure S2). Most TEs were not located in exons or introns, as is obvious from Figure 4 and shown in detail in Table S3A,B. While on average only 0.01% of TEs overlapped with exons, there was limited overlap between TEs and introns (Figure 4) with an average of 2.0–2.1% for all fosmids and 9.8% for the second set of 11 fosmids (Figure S2). The medians for both features were 0 and 0–0.5%, respectively (Figure 4). The minimal overlap between TEs and coding regions is also visible in the sequence features of fosmids, of which 38 examples are visualized in Figure 5. The BUSCO analyses retrieved 23 complete BUSCO genes, of which 19 were single-copy and four were duplicated, and an additional nine fragmented genes; 98.8% of the 2675 searched arthropod BUSCO genes were missing. Thus, in terms of core arthropod genes, the analyzed set of sequences does not comprise particularly gene-poor regions, representing about 1% of core genes, while it constitutes only ~0.35% of total genomic DNA as measured by flow cytometry/DAPI staining.

### 3.3. Additional Domains in R4/Dong and Gypsy Retrotransposons

**Figure 6:**
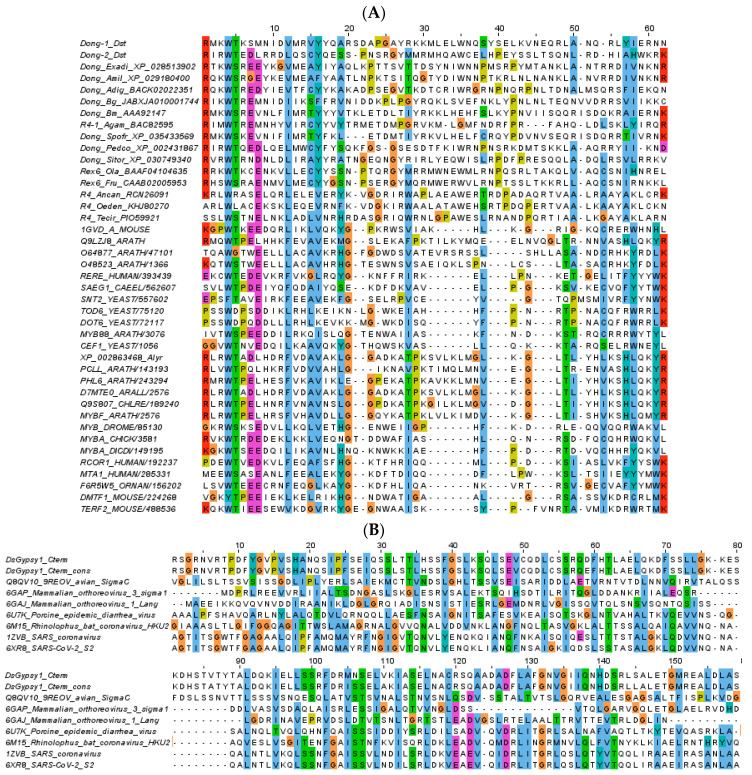
Regions of homology in *R4/Dong* (**A**) and *Gypsy* (**B**) retrotransposons of *D. stevensoni*. Multiple sequence alignments were visualized with Jalview [59]. (A) The alignment includes *Dong1* and *Dong2* (*D. stevensoni*) and related elements from cnidarians (*Exaiptasia diaphana, Acropora millepora, Acropora digitata*), mollusks (*Biomphalaria glabrata*), insects (*Bombyx mori, Anopheles gambiae, Spodoptera frugiperda, Pediculus humanus corporis, Sitophilus oryzae*), fish (*Oryzias latipes, Takifugu rubripes*) and nematodes (*Ancylostoma caninum, Oesophagostomum dentatum, Teladorsagia circumcincta*) with the corresponding accession numbers, followed by a representative selection of Myb-like domains from the PF00249 seed alignment (25 out of 147). (**B**) Structure-based alignment of *D. stevensoni Gypsy1* C-terminus (amino acids 1322-1479 out of 1486) and the central helix domain of the spike (sigma) proteins from reoviruses and coronaviruses identified by HHpred, with the corresponding PDB accession numbers.

While the majority of complete or nearly complete DNA and RNA TEs revealed the expected domain architectures, two retrotransposon families, *R4/Dong* and *Gypsy* (Figure 5B and Figure 6), deserve special mention. Members of the *R4/Dong* clade of non-LTR retrotransposons contain the reverse transcriptase and REL-endonuclease domains and insert into rDNA or into microsatellite targets. However, no recognizable motifs could be previously distinguished at their N-terminus, in contrast to rDNA-specific R2 retrotransposons with a similar REL-endonuclease, which harbor an N-terminal Myb DNA-binding domain [60,61,62,63,64]. We aligned two *Dong* representatives with a full-length ORF from contigs 45 and 333 (Figure 5B) and a set of diverse *R4/Dong*-like elements from cnidarians, mollusks, insects, fish and nematodes, and used HHpred [62] to uncover a highly diverged SANT/Myb-like domain close to the N-terminus (Figure 6A), with the best scores obtained from rDNA-specific *R4* elements of nematodes (94.9% probability hit to PF16282.6 for *Ancylostoma caninum* R4). In the well-studied rDNA-specific *R2* clade, this domain reportedly directs site-specific insertion into rDNA, along with accompanying Zn-finger motifs [63]. Thus, it may be argued that the divergent Myb version found in *Dong*-like elements is similarly responsible for site-specific integration, albeit in the absence of Zn-finger motifs at the N-termini. In *D. stevensoni*, the *Dong* insertion target is represented by (TAA)_n_ repeats, as is the case in most insects, mollusks and cnidarians, and is therefore located outside coding sequences.

The structure of *Gypsy*-like LTR-retrotransposons is similar to that of retroviruses, with the *gag* gene encoding a nucleocapsid and the *pol* gene encoding protease, reverse transcriptase and integrase enzymatic domains [28]. Inspection of the nearly complete *Gypsy_Ds1* on contig 89 (Figure 5B) reveals an atypical 250-aa extension beyond the integrase domain, which typically ends with a GPY/F motif, but may contain an additional chromodomain at the extreme C-terminus. However, in the C-terminal extension of *Gypsy_Ds1 pol*, HHpred identified remote similarity to the trimeric coiled-coil domains of spike proteins from enveloped +ssRNA viruses (coronaviruses) and dsRNA viruses (reoviruses) (Figure 6B) [65,66,67]. Although it also carries a potential furin-like protease cleavage site (RxxR), this extension domain is too short to represent a fully functional *env* (~600 aa), which is responsible for interaction with host membranes during viral entry and egress and is often found in LTR-retrotransposons as the third ORF [68]. Rather, it may be a remnant of an original *env* that was captured from an RNA virus and used for initial horizontal entry into the *D. stevensoni* host. Possible *env*-like ORF3 remnants were found in *Vesta* LTR-retrotransposons in the bdelloid rotifer *Adineta vaga* [69]. However, it is also possible that the C-terminal extension is unrelated to *env*, and the coiled-coil domain could instead be used for interaction of integrase with other proteins.

## 4. Discussion

### 4.1. Comparing Results from the Draft Genome and the Fosmid Library

Here, we show that our results of TE features in a draft genome and the data from a fosmid library perfectly complement each other. We found that estimates of TE diversity, substitution levels and abundance (see Section 4.2 and Section 4.3 for more details) obtained from both genomic resources are in good agreement, validating the observed patterns. At the same time, for detailed examinations of TE insertion sites and testing for possible associations between TEs and telomeres (see Section 4.4.), we could mostly rely on the fosmid library data, as the draft genome of *D. stevensoni* is highly fragmented with 62,118 scaffolds and a N50 of 56 kb [45]. Our results can be further validated and extended as soon as the high quality reference genome of *D. stevensoni* becomes available.

### 4.2. TE Diversity and Substitution Levels

Our results show a high diversity of transposons in the genome of the putative ancient asexual ostracod *D. stevensoni* from both fosmid library and draft genome data (Figure 3A,B and Figure 5). Compared to studies in another presumed anciently asexual taxon, bdelloid rotifers, the genome of the ostracod *D. stevensoni* seems to have a higher TE proportion for all major transposon groups. Given the exceptionally low transposable element load in bdelloids, especially for LINE-like elements [10,13,14], this result could be expected. Two species of putative ancient asexual oribatid mites, for which TE data are also available [18], contained similar levels of TE diversity as observed in the current study. In all three groups of putative ancient asexuals, the majority of TEs were DNA transposons (Figure 3A,B and Figure 5; [10,13,14,18]). However, this was not the case for the (younger) asexual water flea *Daphnia* [17,70,71] and for *Meloidogyne* nematodes [72]. The higher abundances of DNA transposons in putative ancient asexuals can probably best be explained by their prevailing transmission mode, which is often horizontal [31,73,74,75], and thus potentially less influenced by long-term loss of meiosis and sexual reproduction. In aquatic habitats where the ostracod *D. stevensoni* and rotifers occur, horizontal transmission of TEs seems to be more common than in terrestrial habitats, because DNA is not exposed to UV or dry air [76].

The low number of nucleotide substitutions in TEs that was observed in the current study from both fosmid and genome data (Figure 3A,B) indicates that the majority of TEs in the genome of *D. stevensoni* have recently been active. Similar patterns were described for the putative ancient asexual bdelloid rotifer *A. vaga* [76]. A study of Bast et al. [18] revealed higher levels of nucleotide substitutions in TEs of oribatid mites, which is inconsistent with recent activity. Since it is unclear if the methodologies of [18] were fully equivalent to the analyses conducted here and to those obtained in [77], these differences will not be further discussed. Active transposition seems generally to be widespread in both animal and plant genomes [30], but uncontrolled copy number increase can be counteracted by genome defense systems, DNA decay and loss, and negative selection against deleterious insertions and rearrangements.

### 4.3. TE abundance

Eukaryotic genomes can differ drastically in their genomic TE content, ranging from a few to over 80% [30]. With an estimate of 19.1% (Figure 3) for all fosmid data, the transposable load in our fosmid DNA sequence data is slightly lower than the estimated TE abundance of *D. stevensoni* at the whole genome level (Figure 3B). This discrepancy can be explained by the fact that the Illumina repeat library showed about 5% higher TE content owing to the addition of more divergent TE regions, which were only partially covered by Censor. Remarkably, the most abundant mariner-like DNA TEs, which are also the shortest and are easily recognizable by the *de novo* identification pipeline in both fosmid and Illumina data, show almost exactly the same abundances of 8.2 and 8.1%, respectively. While final conclusions on the transposon abundance in *D. stevensoni* should be deferred until high quality, genome-wide data from long read technologies become available, a transposable load of up to 26% (of known TEs) would be in the range of TE loads in other arthropods. In insects, TE abundances range from 6 up to 58% [78]. In aquatic crustaceans other than ostracods, such as crabs and shrimps, repeats and TEs can make up between 50.4 and 57% [79,80,81] of the genome, although TE content from 7.4 to 12.9% was reported for different assemblies of *Daphnia pulex* [17,70,71]. In other, probably younger, asexuals, TE content at the genome-wide scale can exceed 50%, as for example in *Meloidogyne* root-knot nematodes [72]. 

### 4.4. TE Insertion Sites

One possible cause for the relatively high TE content of *D. stevensoni* in our sequence data could be their preferred genomic locations. Through partial selection of fosmids containing transposons, we might have sequenced some TE islands in the genome of *D. stevensoni* outside of coding regions. Our results detected up to 19.4% exons in individual fosmids but only an average overlap of up to 0.01% between exons and TEs and up to 2.1% between introns and TEs in the boxplot (Figure 4). The low amount of observed overlap supports this point of view, and is illustrated for 38 selected fosmids in Figure 5A–F. These observed patterns would largely be consistent with predictions [30] on the predominantly neutral character for the bulk of TE insertions but will require genome-wide data for confirmation. The available Illumina draft genome of *D. stevensoni* is too fragmented [45] to further test these assumptions.

Both the draft genome of *D. stevensoni* and the sequenced fosmids seem to harbor recently active transposons, as supported by the estimated low substitution levels indicating recent activity (Figure 3) and from overlapping TE-rich regions of fosmid contigs (see examples in Figure 5A–F). These indications for relatively recent transposition, together with the low number of insertions within exons (Figure 4), at least partly fit the dichotomous pattern described from fungal transposons [82], where young TEs were not located inside genes, while older TEs were found both inside and outside of coding regions. It is also possible that the sequenced putative TE islands of *D. stevensoni* in the fosmid dataset could come from pericentromeric regions, given that we did not sequence any fosmids containing telomeres. Other studies on humans [83] and rice [84], for example, found a higher abundance of TEs near centromeres. The lack of LINE-like TEs being associated with telomeres in *D. stevensoni* in fosmid sequence data also indicates that there has been no co-option of the investigated TEs for telomeric functions, as in *Drosophila* [85] or bdelloid rotifers [86]. 

### 4.5. Assessing the Impact of Putatively Ancient Asexual Reproduction on TE Landscapes in Non-Marine Ostracods 

Following one of the predictions outlined in the introduction, long-term asexuality is expected to increase the transposable element load because of reduced efficiency of selection for the removal of deleterious mutations and transposons in finite populations [4,5,6]. Our results are consistent with this hypothesis. It is possible that asexual darwinulid ostracods inherited relatively high transposable loads from their sexual ancestors when abandoning sexual reproduction, which could go back as far as 200 myr ago [37] for the whole Darwinulidae and to 25 Mya in the species *D. stevensoni* [38]. Glémin et al. implied that it might take a long time for asexuals to purge TEs from their genomes [25]. Our results indicate that even millions of years have not been sufficient for *D. stevensoni* to efficiently purge its TEs.

Unrestrained TE proliferation should eventually drive asexual lineages to extinction, unless asexual hosts can tightly control TE copy numbers [10,11,12], with putative ancient asexual bdelloids as the most striking example [10,13,14]. Eukaryotic hosts have developed a wide arsenal of molecular defense mechanisms against TEs [29,87], including for example siRNAs and piRNAs [88], and chromatin-based pathways [89]. The relatively high TE content and diversity in *D. stevensoni* could imply that some of these defense mechanisms are lacking or are less efficient, leading to the accumulation of transposons when these are not removed [25]. This explanation seems more likely than the lack of time for purging TEs from sexual ancestors. Alternatively, the insertion pattern of TEs in *D. stevensoni,* as suggested by our preliminary observations, may be biased towards non-coding regions, and could therefore have less deleterious effects on the host. However, the observed lack of insertions into exons as seen in the fosmid data is most likely caused by selection against genic insertions. Finally, certain TEs, such as *Dong*, display insertion specificity that directs them away from coding regions. 

Another factor that can potentially influence transposon abundance is the presence of bacterial endosymbionts. Kraaijeveld et al. suggested that the higher copy numbers of *gypsy* transposons in asexual wasps as compared to sexual relatives [23] could partly be due to the feminizing endosymbiont *Wolbachia,* which is only present in asexuals. The *Cardinium* endosymbionts, which could have similar effects on their hosts as *Wolbachia*, have recently been described in non-marine ostracods including *D. stevensoni* [90], but their potential effect on transposable element loads in asexual ostracods remains to be investigated.

## 5. Conclusions

Our results, based on an Illumina draft genome [45] and parts of a fosmid library, indicate that asexual reproduction in the ostracod *D. stevensoni* did not substantially reduce its transposable load, as may have been the case in other asexual taxa [3,10,11,13,15,16,73]. On the contrary, it rather seems that this ostracod species is another example of an asexual that is less efficient in removing TEs. Whether this is due to the loss or dysfunction of molecular defense mechanisms controlling TE abundance, the absence of methylation, preferred insertion outside of coding regions, or other factors, remains to be investigated in future studies when comparative genomic and transcriptomic data of high quality for this and other ostracod species become available. The absence of sex in darwinulid ostracods has been estimated from fossil data to be as old as 25 myr (*D. stevensoni* [38]) up to 200 myr (the entire post-Palaeozoic family Darwinulidae, using *Alicenula* as a proxy [37]), and many sexual and asexual darwinulids became extinct after the mass extinction at the Permian–Triassic border [91]. The fact that about 30 putative ancient asexual darwinulid species are still present today suggests that TE proliferation has not driven all of these species to extinction, as might be expected [11,12]. Comparisons with TE abundance in sexual ostracods and younger asexual species are required to test whether the observed patterns of TE abundance and diversity are general features related to the old age of Ostracoda of more than 400 myr [92] or to the ancient asexuality of the post-Palaeozoic Darwinulidae. 

## Data Availability

Fosmid sequence data have been submitted to NCBI, accession numbers MW583466-MW583569.

## Supplementary Materials

The following are available online at https://www.mdpi.com/2073-4425/12/3/401/s1, Figure S1: Box plot of size distribution of analyzed contigs from the fosmid library. Figure S2: Comparison of sequence features between three groups of fosmids. Table S1: Probes used for hybridization and detection of fosmids containing TEs or telomeres. Table S2: Hybridization signal of selected fosmids for sequencing. Table S3A: Contig sequence features. Table S3B: Fosmid sequence features. Table S4: Overview of possible overlap between contig ends. Table S5: Results of TE identification with Censor.
