# Supplementary material for: A Survey of Transposon Landscapes in the Putative Ancient Asexual Ostracod Darwinula stevensoni
Source: Genes (Basel). 2021 Mar 11;12(3):401. doi: 10.3390/genes12030401 (PMC7998251; doi:10.3390/genes12030401)
Supplement: Supplementary file 1 [file genes-12-00401-s001.zip › supplementary material_resubmission/original_manuscript_with track changes.pdf]

genes

MDPI

Article

A survey of transposon landscapes in the putative ancient asexual ostracod *Darwinula stevensoni*

Isa Schön[1,2*$], Fernando Rodriguez[3a$], Matthew Dunn[3b], Koen Martens[1,4], Michael Shribak[3c] and Irina Arkhipova[3d]

[1] Royal Belgian Institute of Natural Sciences, OD Nature, Freshwater Biology, Vautierstraat 29, B-1000 Brussels, Belgium; ischoen@naturalsciences.be
[2] University of Hasselt, Researchgroup CMK, Diepenbeek, Belgium; ORCID 0000-0001-9269-6487
[3] Marine Biological Laboratory, MBL Street 7, Woods Hole MA 02453, USA
[a] ORCID: 0000-0003-4044-8734; frodriguez@mbl.edu
[b] mjdunn@umass.edu
[c] ORCID: 0000-0002-5849-6294; mshribak@mbl.edu
[d] ORCID: 0000-0002-4805-1339; iarkhipova@mbl.edu
[4] University of Ghent, Dept Biology, Ghent, Belgium; ORCID: 0000-0001-8680-973X; darwinula@gmail.com; ORCID: 0000-0001-8680-973X
* Correspondence: ischoen@naturalsciences.be
[$] Co-first

Received: date; Accepted: date; Published: date

Abstract: How asexual reproduction shapes transposable element (TE) content and diversity in eukaryotic genomes remains debated. Here, we performed an initial survey of TE load and diversity in the putative ancient asexual ostracod *Darwinula stevensoni*. We examined long contiguous stretches of DNA in clones selected from a genomic fosmid library, totaling about 2.5 Mb, and supplemented these data with results on TE abundance and diversity from an Illumina draft genome. In contrast to other TE studies in putatively ancient asexuals, which revealed relatively low TE content, we found that at least 19% of fosmid DNA sequences and 26% of the genome corresponded to known transposons. We observed a high diversity of transposon families, including LINE, gypsy, PLE, *mariner/Tc, hAT, CMC, Sola2, Ginger, Merlin, Harbinger,* MITEs and helitrons. The predominantly low levels of sequence diversity indicate that many TEs are or have recently been active. No correlation was found between telomeric repeats and non-LTR retrotransposons present near telomeres in the fosmid data as in other taxa. We found that most TEs in the fosmid data were located outside of introns and almost none were found in exons. We also report an N-terminal Myb/SANT-like DNA-binding domain in site-specific *R4/Dong* non-LTR retrotransposons. Although initial results on transposable loads will need to be verified at a genome-wide scale with high quality draft genomes, this study provides important first insights into the dynamics of TEs in putative ancient asexual ostracods.

Deleted: In the absence of a high-quality whole-genome draft, w…

Deleted: .1

Deleted: M

Keywords: transposable elements; retrotransposons; DNA transposons; crustaceans; asexuality

1. Introduction

The influence of reproductive mode on transposon content and distribution in eukaryotic genomes remains a subject of debate as it seems to be shaped by several evolutionary forces acting in opposite directions. Sexual reproduction is expected to greatly facilitate spread of vertically transmitted transposons in populations [1], as has been experimentally shown in yeast (see for example [2] or [3]).

All asexuals originate from sexual ancestors. If they inherited transposons, the absence of meiotic recombination should lead to reduced efficacy of selection and accumulation of deleterious mutations [4] and transposons, at least in finite populations [5,6]. Empirical evidence for these predictions comes from non-recombining sex chromosomes e.g. [7] and other non-recombining parts of the genome [8,9]. Such unrestrained TE proliferation should eventually drive asexual lineages to extinction unless asexual hosts can keep TE copy numbers under tight control [10-12], with putative ancient asexual bdelloids as the most striking example [10,13,14] of having reduced numbers of vertically transmitted retrotransposons while they still harbor some DNA transposons that can be horizontally transmitted. In line with these predictions, sexual *Daphnia pulex* have higher loads of DNA transposons [15], LTR retrotransposons [16] or more insertion polymorphisms of transposons [17] than their asexual counterparts. Comparative genomic studies on asexual and sexual arthropods [18], nematodes [19], evening primroses [20] and green algae [21], however, did not show any significant effect of the reproductive mode on transposon content and evolution. Other genomic studies reported higher transposon loads in asexuals as for example for root-knot nematodes [22] or parasitic wasps [23].

These contrasting results can partly be explained by different lineage ages [24]. Purging of transposons in asexuals can take a very long time, and transposons are expected to accumulate if they cannot be removed [25]. Theoretical studies [26,27] and an ever-increasing number of genomic studies from different host organisms [28,29] suggest that additional factors besides the reproductive mode will also influence transposon diversity and load, such as initial transposon load in sexual ancestors, DNA methylation, population size [30], environmental fluctuations [27], strengths of selection and drift [19], and molecular defense mechanisms against transposable elements. DNA transposons are more frequently transmitted horizontally (as in the example of insects; [31]) than retrotransposons and are expected to be less affected by the reproductive mode.

To better understand transposon evolution, their molecular characteristics and biological effects, additional *in vivo* and *in vitro* studies are thus required [28], especially from non-model organisms. The continuous increase of genomic data has also revealed the extent of lineage-specific transposon diversities, which further increases the methodological challenges of analyzing these elements [28] and provides additional motivation for studying them in a wide range of organisms.

The ostracod family Darwinulidae is one of the few examples of putative ancient asexual animals [32-35], to which bdelloid rotifers also belong [36]. Fossil data indicate that some darwinulids might have been asexual for 200 million years [37], and the type species of this family that is investigated here, *Darwinula stevensoni* (Figure 1), for about 25 million years [38]. There is only one study on transposable elements of darwinulid ostracods describing novel LINE-like retroelements [39], and long, contiguous genome assemblies from these ostracods are not yet available.

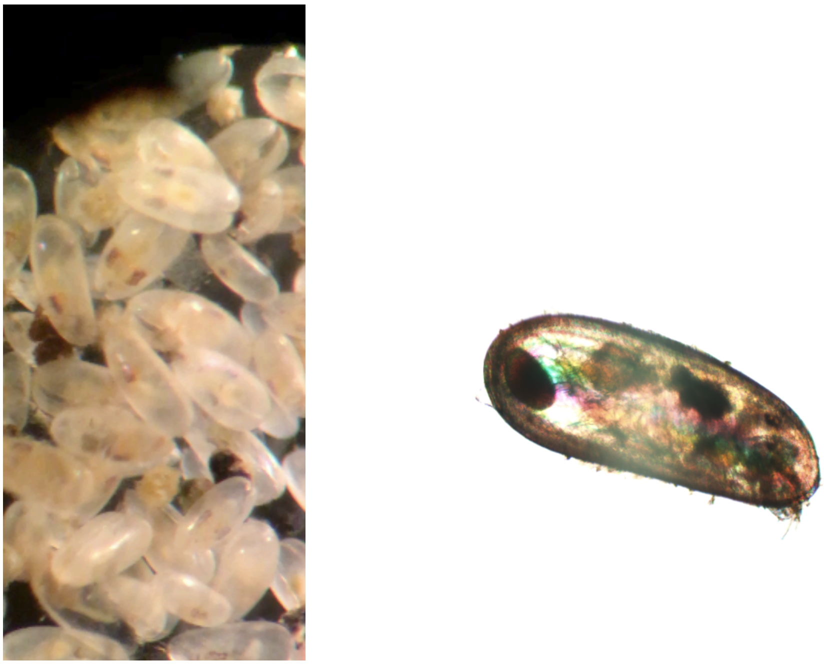

Figure 1. *Darwinula stevensoni*. a. A sample of multiple individuals of *Darwinula stevensoni*. Taken by Jeroen Vendericks. b. Lateral view of the carapace of an individual *Darwinula stevensoni*. This picture was taken with the polychromatic polarization microscope [40] with a 4x objective lens and a DP73 camera. The total length of the animals is around 800 µm. In the left corner, an embryo in the brooding pouch is visible.

Here, we used long DNA sequences of *D. stevensoni* based on selected clones of a genomic fosmid library, totaling around 2.5 Mb, for addressing four aims: (1) To gain initial insights into TE content, diversity and activity in darwinulid ostracod genomes and confirm these preliminary results with data from an Illumina draft genome of *D. stevensoni* ; (2) Examine possible links of TEs with telomeres; (3) Compare the location of TEs to coding gene regions (CDS); (4) Assess the possible impact of anciently asexual reproduction on TE landscapes of non-marine ostracods. We observed a high diversity of TEs in the putative ancient asexual ostracod *D.*

Formatted: Font: Italic

stevensoni, and a high prevalence of 19-26% in the surveyed fosmid and draft genome DNA sequence data. Most TEs were located outside of coding regions, had no link to telomeres and showed evidence of recent activity. Our results provide first indications that putative ancient asexual ostracods might not be able to efficiently purge TEs from their genomes.

2. Materials and Methods

2.1. Construction, screening and sequencing of a genomic fosmid library

A genomic fosmid library *of D. stevensoni* was constructed at Clemson University (USA) from 1000 pooled individual ostracods, sampled from the monoclonal Belgian population in Hollandersgatkreek, because of the small size of these ostracods (L= c 0.8 mm - Figure 1a & b). Ostracods were first incubated for ten days in pure water to evacuate any possible contaminants from the gut. High molecular weight DNA was isolated, randomly fragmented, end-repaired, phosphorylated, and size selected by pulsed-field gel electrophoresis. Size-selected fragments were ligated with the linearized dephosphorylated fosmid vector pCC2FOS (NovoPro) and then packaged by lambda packing extracts and plated on T1-phage resistant *E. coli*. A total of 18,432 recombinant colonies were randomly picked with a Genetix Q-bot robot and stored as individual clones at -80°C in 384-well microtiter plates. Fosmids have an average insert size of 35 to 45 kbp, as was confirmed by randomly sampling 48 clones. Preliminary evaluation of *D. stevensoni* from the same Belgian population with flow cytometry and DAPI staining revealed a genome size of 0.86 to 0.93 pg, equaling 840 to 900 Million bp (Paczesniak et al., unpubl. data). Karyological studies have shown that *D. stevensoni* nuclei contains 22 dot-like chromosomes [41] that cannot be visually grouped into homologous pairs, making it impossible to infer the ploidy level of *D. stevensoni* from cytological observations.

To identify fosmids containing either TEs or telomeres, specific overgo probes for LINE-like and mariner-like elements and telomeric repeats were developed (see Table S1 for details) from published data on two LINE-like elements of *D. stevensoni* [39] and unpublished data on TEs from non-marine ostracods, which had been acquired with PCR walking and Sanger sequencing using the general primers of [10]. Probes for telomeres were based on the universal arthropod telomeric repeat (TR) with the pentameric unit TTAGG [42]. We also screened for fosmids containing single-copy nuclear genes of *D. stevensoni* [43].

High density colony filters from the entire fosmid library were produced using a Genetix Q-bot in a 4x4 double-spotted array on GE HealthCare Hybond N+ membranes. Labelled PCR probes were used for hybridization of high-density colony filters and hits were called with Hybdecon v.01. The identity of fosmids having hits for TEs, single copy nuclear genes and/or TRs was assessed. The hybridization experiments with the fosmid library revealed 86 fosmids with positive hits for the mariner probe, 18 for LINE-like *Daphne*, 33 for LINE-like *Syrinx* elements and 40 fosmids with hits for various nuclear genes, respectively (Table S2). A set of

Deleted: more than

Deleted:

Deleted: 5

96 fosmids, of which 13 contained positive hits for chosen TEs (5 Daphne, 5 Syrinx, 3 mariner) and the other 83 for nuclear genes, were selected for further detailed analyses. DNA was extracted and prepared for high-throughput sequencing with the Ion Xpress™ plus gDNA Fragment Library preparation kit and the Ion OneTouch™ 200 Template Kit v2 from Life Technologies. Individual fosmids were sheared, ligated to adapters with barcodes, size selected, pooled and used for an emulsion PCR. Fosmids were sequenced with the Ion PGM™ Sequencing 300 Kit on the Ion Torrent PGM using the Ion 316™ chips. A total of 779M DNA basepairs and 2,827,903 reads were generated, with a median read lengths of 290 bp and maximum lengths of 433 bp. Fosmids were demultiplexed, and quality filtering and assembly conducted with CLC workbench (Qiagen; version 7.5.1) using default parameters.

Another 11 fosmids were selected by hybridization with probes for single nuclear copy genes of potential horizontal origin (see Table S2) as part of the LATTECO project at the CNRGV (INRA facility, Toulouse, France). Sequence identities of the ends of positive fosmids were validated by PCR and Sanger sequencing. For PacBio RS II sequencing, 2 µg of each validated fosmid was tagged with PacBio tags and then pooled. The library was generated with the standard Pacific Biosciences library preparation protocol for 10 kb libraries and sequenced on one SMRT Cell using the P6v2 chemistry following the standard operating procedures of the manufacturer at the NGI (https://ngisweden.scilifelab.se/). Assembly of the PacBio RS II reads followed the HGAP workflow. The SMRT® Analysis (v2.2.0) software suite was used for HGAP implementation. Reads were first de-multiplexed and then aligned using BLASR against "*E. coli* str. K12 substr. DH10B, complete genome". Identified *E. coli* reads and low-quality reads (read quality <0.80 and read length <500 bp) were removed. Filtered reads were then preassembled to generate long and highly accurate sequences. To perform this step, we separated the smallest and longest reads (e.g. >11 kbp) in order to correct read errors by mapping the smallest to the longest reads. Obtained sequences were filtered against vector sequences, and sequences were assembled into draft assemblies with the Celera assembler. As the final step of the HGAP workflow, "polishing" with Quiver significantly reduced remaining insertions and deletions and base substitution errors, resulting in high quality assemblies of a single contig per fosmid.

We analysed 341 contigs with a minimum length of 100bp for further analyses. We also conducted BLAST searches [44] with all contigs to each other to identify potentially overlapping redundant regions. The content of matching regions was further checked manually. If these included identical transposons, we considered them as evidence for recent transposition and kept the data. If (short) contigs fully matched other contigs, they were excluded. Likewise, short overlaps at the 5′ or 3′ end of fosmid sequences were also excluded if they showed more than 94% overlap with other contigs. In the absence of a phased reference genome, we can however not be certain if overlapping contigs originate from the same chromosome or from its homolog, as the allelic divergence is expected to be low, especially if the overlap is relatively short. To still account for possible bias from overlapping contig ends,

Deleted: some

Deleted: most

Formatted: Highlight

Formatted: Highlight

we are providing ranges for contigs and fosmid total lengths and estimates of exon, intron and TE content below.

All sequence data have been submitted to the GSS of ncbi; accession number XXXXX.

2.2. *Illumina draft genome of D. stevensoni*

Besides the fosmid DNA sequence data, we also analyzed a recently published Illumina draft genome (European Nucleotide Archive accession number PRJEB38362) of *D. stevensoni* [45} for its TE content and diversity. This draft genome was assembled from DNA extractions of a single female, followed by Whole Genome Amplification to provide sufficient material for the preparation of three 2x125bp paired-end libraries (average insert sizes of 250-300, 550 and 700bp), and two mate-pair libraries (average insert sizes of 3000 and 5000bp), respectively, which were sequenced on an Illumina HiSeq 3000 system. The assembly has a size of 382.1 Mb, an N50 of 56.4 kb, an arthopod BUSCO score of 93.7% (complete single copy genes) and consists of 62,118 scaffolds [45].

2.3 *De novo identificiation of ostracod TEs*

We used the REPET package with default settings [46,47] for *de novo* TE identification and annotation of fosmid and draft genome data in three steps: detecting repeated sequences and potential TEs, clustering of these sequences, and generating consensus sequences for each cluster. Consensus sequences were classified following Wicker's TE classification [48] and transposons were grouped by families. Repeat Masker [49] was applied for TE classification and plot building, using the local fosmid and genomic libraries of *D. stevensoni* from REPET. We constructed TE landscape divergence plots to evaluate the frequencies of different TE families in our data set and estimate the Kimura substitution level of each TE family with adjusted CpG as a measure of TE activity over time. We also translated all fosmid DNA sequences and used the translated data in Censor (https://www.girinst.org/censor/) to reveal non-multicopy TEs and to classify TEs at the amino-acid level.

2.4. *Assessing insertion sites of TEs from fosmid data*

For obtaining preliminary information on the genomic location of TEs, we compared hybridization signals between fosmids with positive hits for telomeres and TEs. To assess if TEs were found in coding or non-coding genomic regions, we used our custom TE library with Repeat Masker to identify and soft-mask all TEs. The masked DNA sequence data were then used for gene predictions with Augustus [50] with *Drosophila melanogaster* as species parameter. In regions being identified as coding regions in the sequenced fosmids, the lengths of exons and introns were calculated from the exact locations to estimate the overall frequency of exons and introns in the DNA sequence data. To identify possible overlap of TEs and exons, we used BEDTools v2.29.2 [51] to compare the exact exon and intron locations in

Deleted: (to be completed during review
Formatted: Highlight
Deleted: *De novo identificiation of ostracod TEs*¶
Formatted: Font: Italic
Deleted: 45
Deleted: 46
Deleted: 47
Deleted: 48
Deleted: y
Formatted: Highlight
Commented [MOU1]: Fernando, please confirm that we only did this for the fosmid data.
Deleted: 3
Deleted: 49
Commented [MOU2]: Fernando, if you could please add the software here?
Formatted: Highlight
Deleted: 50

each fosmid with partial and complete TE locations as identified by Censor at the amino-acid level.

We calculated the frequency of introns and exons, TEs and overlap with exons and introns, respectively, per fosmid and visualized these as boxplots in ggplot2 [52] in R [53]. For selected fosmids, we used the output files of gene predictions with Augustus and TE identification by Censor to draw the positions of transcripts, exons, introns and TEs with Circos [53].

2.5. Estimating single copy gene content in fosmid sequence data

To assess the representativity of selected fosmids with regard to coding sequences, we conducted Benchmarking set of Universal Single-Copy Orthologs (BUSCO) v3.0.2 analyses [55] of all fosmid sequence data using Arthropoda_odb9 as reference database for single copy ortholog genes [56].

2.6. Search for remote homologies

To investigate the N-terminal domain in *R4/Dong* elements, we assembled a dataset of 5′-complete ORFs which included phylogenetically diverse additional sequences from GenBank identified by BLAST (accession numbers shown in Fig. 6), aligned with MUSCLE [57], extracted the N-terminal part upstream of the reverse transcriptase domain, and used the multiple sequence alignment as a query on the HHpred server [58] with default settings. The C-terminal extension of *DsGypsy1* had no detectable homologs and was used as a standalone query. The seed alignment for PF00249 (*Myb_DNA-binding*) was downloaded from PFAM (http://pfam.xfam.org/). Structure-based alignments obtained with HHpred were visualized with Jalview v.2.11.1.3 [59] using the Clustal color scheme.

3. Results

3.1. TE diversity, substitution levels and abundance

The longest 341 contigs from 95 fosmids (Figure S1), providing a total of 2,39 Mbp-2,55 Mbp with an average length of 7,657bp-7,472 bp and a median of 4,242-4390bp (Table S3A & S3B) were further analyzed for TE and gene content (Figure 2). Details on potentially overlapping contigs are provided in Table S4.

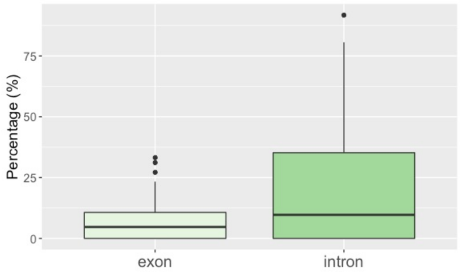

Deleted: 51
Deleted: 52
Deleted: *4*
Deleted: 54
Deleted: 55
Deleted: *5*
Deleted: 56
Deleted: 57
Deleted: 58
Deleted: *<object>*
Deleted: 2

Figure 2: **Box plots of intron and exon abundance.** The frequency of introns and exons were calculated as % of total fosmid lengths. Boxes contain the interquartile range from the 25th to the 75th percentile, the horizontal line indicates the median, and vertical lines minimum and maximum distributions of the data. Outliers are shown by dots.

Our analyses revealed a high diversity of TEs in the selected clones from the fosmid library of *D. stevensoni* and the Illumina draft genome [45] including LINE-like retrotransposons, LTR retrotransposons, various cut-and-paste DNA transposons and Helitrons (Figure 3). DNA transposons were best represented at 8.9% in the fosmids and 11.8% in the Illumina data, and belonged to Tc/mariner (most abundant at 8% in both datasets), hAT, Ginger, Merlin, Harbinger, and CMC-like DNA transposons, plus some uncharacterized DNA transposons. Similarly, for retrotransposons we found five major superfamilies of LINE-like elements (Jockey/I, CR1, L2, R4, RTE) with a total abundance of 4.2% and 2.8% in the fosmid and Illumina data, respectively, as well as gypsy-like (1%), Penelope-like elements (0.3%) and other unassigned retrotransposons (<0.1%) in the fosmids. Additionally, 4.2% of the sequence data were classified as non-LTR by Censor (Table S5), while Helitrons constituted 0.3% of the fosmid and 1.5% of the genomic sequence data. The high diversity of TEs in the fosmid data is also illustrated in 38 selected contigs containing mariner-1 (Figure 5a) and mariner-2 DNA TEs (Figure 5c, d), a mixture of DNA, LINE-like and LTR TEs (Figure 5b), and LINE-like RTE (Figure 5e) and CR1 (Figure 5f) TEs.

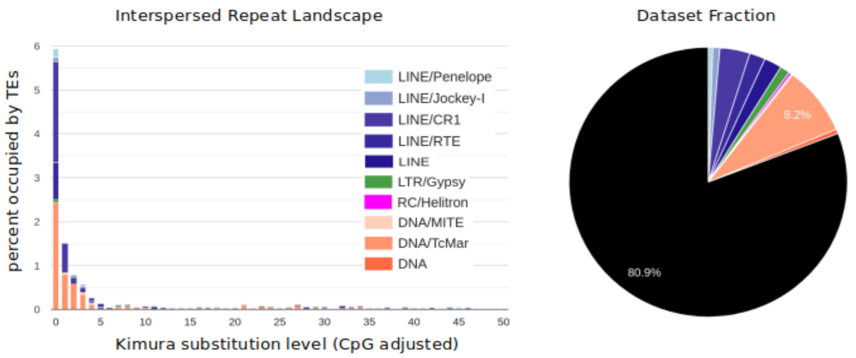

Deleted: of fosmid size (a) and

Deleted: of introns and exons

Deleted: (b). The size range of fosmids is provided in basepairs (bp). ...

Deleted: ,

Deleted:

Deleted: .2

Deleted: , even though only the mariner probe was used for selection (Table S1 and S2)

Formatted: Highlight

Commented [MOU3]: Irina, could you please advise if we should also report these numbers from the genome data as they are not visible in new Figure 3b or leave them out?

Deleted: DNA

Formatted: Highlight

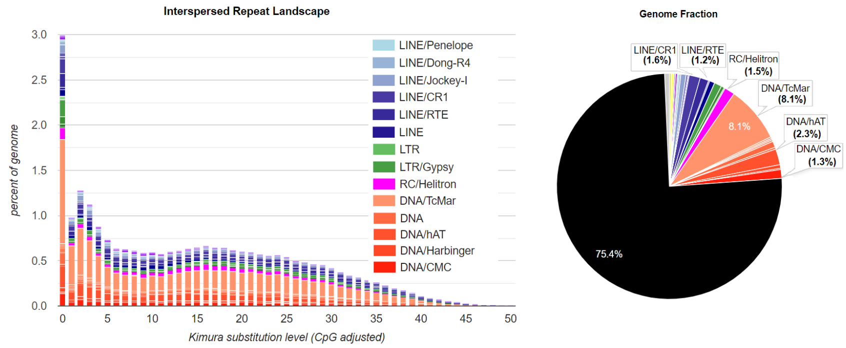

Figure 3: **Landscape divergence plots (left hand side) and genome occupancy by known TEs (right hand side) in *D. stevensoni*.** Divergences were calculated as Kimura substitution levels with adjusted CpG. Genome fraction of TEs was calculated after merging Repeat Masker and Censor outputs. (a, top) TEs in fosmid DNA sequence data. Genome fraction of TEs was calculated after merging Repeat Masker and Censor outputs. (b, bottom) TEs in the preliminary draft genome assembly [45]. The plot was constructed using the REPET library obtained with the Illumina assembly. The pie chart shows genome occupancy for TE categories occupying more than 1% of the genome.

The distribution of Kimura substitution levels on the TE divergence plot was negatively logarithmic in both data sets (Figure 3a & 3b), with most TEs having low substitution levels and only a few TEs showing high levels of substitution, indicating that most copies originated from relatively recent transposition events. In total, 19.1% of all fosmid DNA sequence data (Figure 3a) and 25.6% of the draft genome (Figure 3b) comprised (known) TE sequences. Repeat Masker and Censor estimated the median frequencies of TEs per fosmid as 8.6%-9.4% and 11.7%-12.8%, respectively, and average frequencies of 16.6%-17.5% and 16.0%-16.7%, respectively (Figure 4 and Table S3B). Average TE abundance estimated at the contig level was similar, with 13.7%-14.7% (Repeat Masker) and 16.5%-16.9% (Censor; Table S3A).

Deleted: fosmid DNA sequence data of

Deleted:

Formatted: Font: Bold

Formatted: Indent: First line: 0 cm

Deleted: (Figure 3)

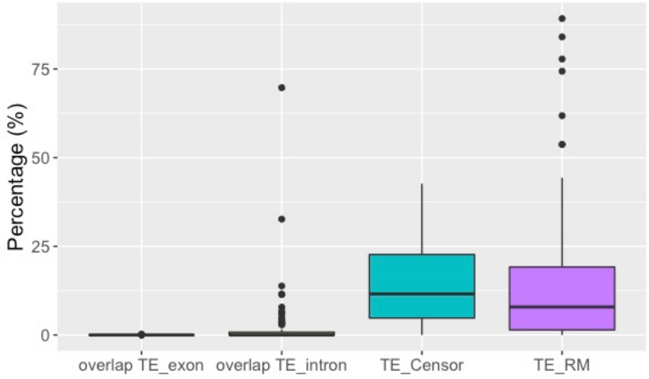

Figure 4: **Box plots of TE abundance and overlap of TEs with exons and introns in fosmid data.** TEs were identified with Censor from translated fosmid DNA sequences. All frequencies were calculated as % of total fosmid lengths. Boxes contain the interquartile range from the 25th to the 75th percentile, the horizontal line indicates the median, and vertical lines minimum and maximum distributions of the data. Outliers are shown by dots.

3.2. TE insertion sites in fosmids and their relationship to telomeres and coding regions

When comparing hybridization signals, there was no overlap between fosmids with a signal for telomeres and fosmids that contained TEs. With regard to coding regions, fosmids contained on average 6.4%-6.7% exons and 20.1% introns, with a maximum of 91.2% introns (Figure 2).

The second set of 11 fosmids that had been selected with probes for nuclear genes contained more exons and introns than the other fosmids (Figure S2). Most TEs were not located in exons or introns, as is obvious from Figure 4 and shown in detail in Tables S3A & B. While on average only 0.01% of TEs overlapped with exons, there was limited overlap between TEs and introns (Figure 4) with an average of 2.0%-2.1% for all fosmids and 9.8% for the second set of 11 fosmids (Figure S2). The medians for both features were 0 and 0-0.5%, respectively (Figure 4). The minimal overlap between TEs and coding regions is also visible in the sequence features of fosmids, of which 38 examples are visualized in Figure 5. The BUSCO analyses retrieved 23 complete BUSCO genes, of which 19 were single-copy and four duplicated, and an additional nine fragmented genes; 98.8% of the searched 2675 arthropod BUSCO genes were missing. Thus, in terms of core arthropod genes, the analyzed set of sequences does not comprise particularly gene-poor regions, representing about 1% of core genes, while it constitutes only ~0.35% of total genomic DNA as measured by flow cytometry/DAPI staining.

3.3. Additional domains in R4/Dong and Gypsy retrotransposons

While the majority of complete or nearly complete DNA and RNA TEs revealed the expected domain architectures, two retrotransposon families, *R4/Dong* and

Deleted: 1

Deleted: seen

Deleted: in

Deleted: 1

Deleted: six

Gypsy (Figures 5b and 6), deserve special mention. Members of the R4/Dong clade of non-LTR retrotransposons contain the reverse transcriptase and REL-endonuclease domains and insert into rDNA or into microsatellite targets. However, no recognizable motifs could be previously distinguished at their N-terminus, in contrast to rDNA-specific *R2* retrotransposons with a similar REL-endonuclease which harbor an N-terminal Myb DNA-binding domain [60-64]. We aligned two *Dong* representatives with a full-length ORF from contigs 45 and 333 (Figure 5b) and a set of diverse *R4/Dong*-like elements from cnidarians, mollusks, insects, fish and nematodes, and used HHpred [62] to uncover a highly diverged SANT/Myb-like domain close to the N-terminus (Figure 6a), with the best scores obtained from rDNA-specific *R4* elements of nematodes (94.9% probability hit to PF16282.6 for *Ancylotoma caninum*). In the well-studied rDNA-specific *R2* clade, this domain reportedly directs site-specific insertion into rDNA, along with accompanying Zn-finger motifs [63]. Thus, it may be argued that the divergent Myb version found in *Dong*-like elements is similarly responsible for site-specific integration, albeit in the absence of Zn-finger motifs at the N-termini. In *D. stevensoni*, the *Dong* insertion target is represented by $(TAA)_n$ repeats, as is the case in most insects, mollusks and cnidarians, and is therefore located outside coding sequences.

The structure of *Gypsy*-like LTR-retrotransposons is similar to that of retroviruses, with the *gag* gene encoding a nucleocapsid and the *pol* gene encoding protease, reverse transcriptase and integrase enzymatic domains [28]. Inspection of the nearly complete *Gypsy_Ds1* on contig 89 (Figure 5b) reveals an atypical 250-aa extension beyond the integrase domain, which typically ends with a GPY/F motif, but may contain an additional chromodomain at the extreme C-terminus. However, in the C-terminal extension of *Gypsy_Ds1 pol*, HHpred identified remote similarity to the trimeric coiled-coil domains of spike proteins from enveloped +ssRNA viruses (coronaviruses) and dsRNA viruses (reoviruses) (Figure 6b) [65-67]. Although it also carries a potential furin-like protease cleavage site (RxxR), this extension domain is too short to represent a fully functional *env* (~600 aa), which is responsible for interaction with host membranes during viral entry and egress and is often found in LTR-retrotransposons as the third ORF [67]. Rather, it may be a remnant of an original *env* which was captured from an RNA virus and used for initial horizontal entry into the *D. stevensoni* host. Possible *env*-like ORF3 remnants were found in *Vesta* LTR-retrotransposons in the bdelloid rotifer *Adineta vaga* [69]. However, it is also possible that the C-terminal extension is unrelated to *env*, and the coiled-coil domain could instead be used for interaction of integrase with other proteins.

Deleted: 59
Deleted: 63
Deleted: 61
Deleted: knuckle
Deleted: 62
Deleted: knuckle
Deleted: 64
Deleted: 66
Deleted: 68

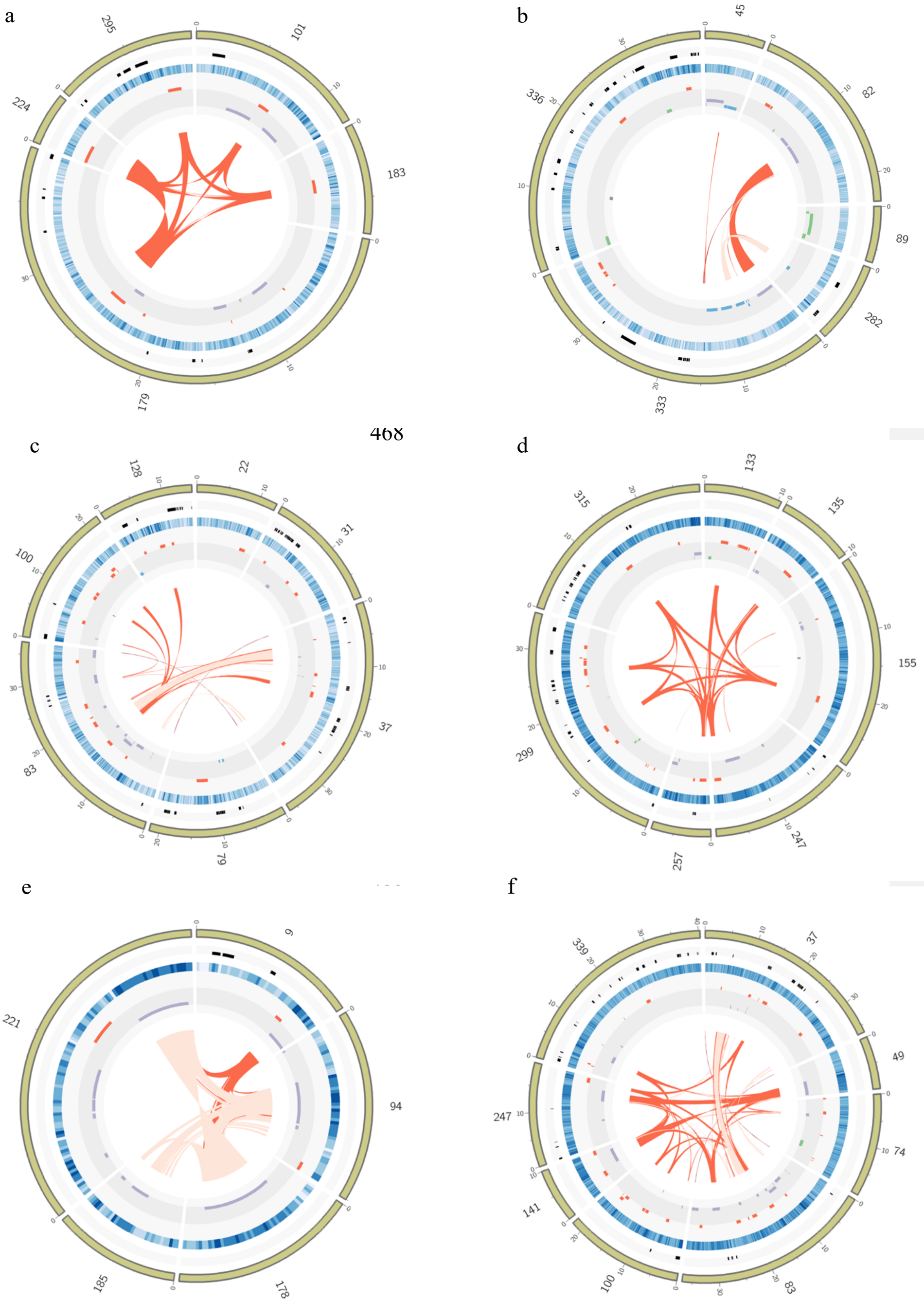
a
b
c
d
e
f

Figure 5: **Sequence features of representative fosmid contigs.** Features of contigs (marked outside in yellow) are plotted on tracks and their identities indicated as coloured boxes in the figure legend. Shades of red represent % identity from BLAST searches (dark color 100%, intermediate 90-99%, and light color 80-89%), indicating recent transposition (see also Table S4). Large numbers on the outside indicate contigs; smaller numbers, the position in kb. Translated TEs were identified with Censor; transcripts, exons and introns were predicted with Augustus; and all features visualized with Circos. For further details on contigs, see Table S3A.
5a: Contigs Ds_ctg224, Ds_ctg295, Ds_ctg101, Ds_ctg183 & Ds_ctg179 containing mariner-1 DNA TEs. 5b: Contigs Ds_ctg 45, Ds_ctg 82, Ds_ctg 89, Ds_ctg 282, Ds_ctg 333 & Ds_ctg 336 with various TEs including DNA (mariner), LINE (PLE and CR1), and LTR (gypsy). 5c and 5d: Contigs Ds_ctg22, Ds_ctg31, Ds_ctg37, Ds_ctg79, Ds_ctg83, Ds_ctg100 & Ds_ctg128 and Ds_ctg133, Ds_ctg135, Ds_ctg155, Ds_ctg247, Ds_ctg257, Ds_ctg299 & Ds_ctg315, respectively, displaying abundant mariner-2 TEs with high sequence similarities. 5e and 5f: Contigs containing LINE-like TEs, including contigs Ds_ctg9, Ds_ctg94, Ds_ctg178, Ds_ctg185 and Ds_ctg221 containing RTE (Fig. 5e) and contigs Ds_ctg37, Ds_ctg49, Ds_ctg74, Ds_ctg83, Ds_ctg100, Ds_ctg141, Ds_ctg247 & Ds_ctg339 containing CR1 (Fig. 5f) TEs.

a

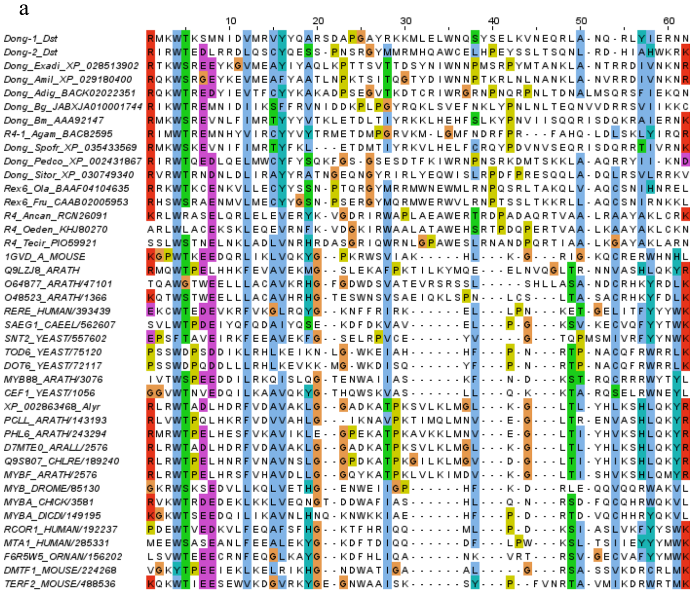

b

Formatted: Highlight

Formatted: Highlight

Deleted: showing, in the inward direction, the positions of exons (in black), GC content (in %, light blue), DNA TEs (in red), LINE TEs (in purple), Penelope-like TEs (in blue), LTR TEs (in green) and other TEs (in grey).

Deleted: hown in s

Deleted: is

Deleted: the

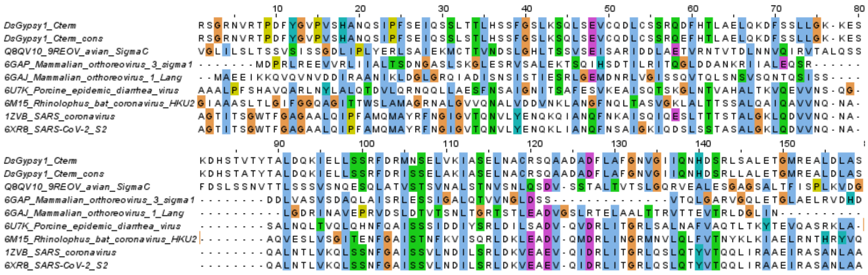

Figure 6: Regions of homology in *R4/Dong* (6a) and *Gypsy* (6b) retrotransposons of *D. stevensoni*. Multiple sequence alignments were visualized with Jalview [59]. 6a: Alignment includes *Dong1* and *Dong2* (*D. stevensoni*) and related elements from cnidarians (*Exaiptasia diaphana, Acropora millepora, Acropora digitata*), mollusks (*Biomphalaria glabrata*), insects (*Bombyx mori, Anopheles gambiae, Spodoptera frugiperda, Pediculus humanus corporis, Sitophilus oryzae*), fish (*Oryzias latipes, Takifugu rubripes*) and nematodes (*Ancylostoma caninum, Oesophagostomum dentatum, Teladorsagia circumcincta*) with the corresponding accession numbers, followed by a representative selection of Myb-like domains from the PF00249 seed alignment (25 out of 147). 6b: Structure-based alignment of *D. stevensoni Gypsy1* C-terminus (amino acids 1322-1479 out of 1486) and the central helix domain of the spike (sigma) proteins from reoviruses and coronaviruses identified by HHpred, with the corresponding PDB accession numbers.

4. Discussion

4.1. TE diversity and substitution levels

Our results show a high diversity of transposons in the genome of the putative ancient asexual ostracod *D. stevensoni* from both, fosmid library and draft genome data (Figure 3 a, b and Figure 5). Compared to studies in another presumed anciently asexual taxon, bdelloid rotifers, the genome of the ostracod *D. stevensoni* seems to have a higher TE proportion for all major transposon groups. Given the exceptionally low transposable element load in bdelloids, especially for LINE-like elements [10,13,14], this result could be expected. Two species of putative ancient asexual oribatid mites, for which TE data are also available [18], contained similar levels of diversity as observed in the current study. In all three groups of putative ancient asexuals, the majority of TEs were DNA transposons (Figure 3 a, b and 5; [10,13,14,18]), however this was not the case for the (younger) asexual *Daphnia* [17,70,71] and *Meloidogyne* nematodes [72]. The higher abundances of DNA transposons in putative ancient asexuals can probably best be explained by their prevailing transmission mode, which is often horizontal [31,73-75], and thus potentially less influenced by long-term loss of meiosis and sex. In aquatic habitats where the ostracod *D. stevensoni* occurs, horizontal transmission of TEs seems to be more common than in terrestrial habitats, because DNA is not exposed to UV or dry air [76].

Deleted: 58

Deleted: Its actual TE diversity is expected to be even higher, given that REPET, which is designed for analyses of whole-genome data, takes into account only transposons with at least three copies, while TEs occurring in the dataset once or twice could be detected only at the protein level by Censor.

Deleted: 69

Deleted: 70

Deleted: 71

Deleted: 72

Deleted: 74

Deleted: 75

The low number of nucleotide substitutions in TEs that was observed in the current study from both fosmid and genome data (Figure 3 a, b) indicates that the majority of TEs in the genome of *D. stevensoni* have been recently active; similar patterns were described for the putative ancient asexual bdelloid rotifer *A. vaga* [76]. The study of Bast *et al*. [18] revealed higher levels of nucleotide substitutions in TEs of oribatid mites, inconsistent with recent activity. Since it is unclear if the methodologies of [18] were fully equivalent to the analyses conducted here and to [77], these differences will not be further discussed. Active transposition seems generally to be widespread in both animal and plant genomes [78], but uncontrolled copy number increase can be counteracted by genome defense systems, DNA decay and loss, and negative selection.

4.2. TE abundance

Eukaryotic genomes can differ drastically in their genomic TE content, ranging from a few per cent to over 80% [79]. With an estimate of 19.1% (Figure 3) for all fosmid data, the transposable load in our fosmid DNA sequence data is slightly lower than the estimated TE abundance of *D. stevensoni* at the whole genome level (Figure 3b). This discrepancy can be explained by the fact that the Illumina repeat library showed about 5% higher TE content due to the addition of more divergent TE regions, which were only partially covered by Censor. While final conclusions on the transposon abundance in *D. stevensoni* should be deferred until high quality, genome-wide data from long read technologies become available, a transposable load of up to 26% (of known TEs) would be in the range of TE loads in other arthropods. In insects, TE abundances range from 6 up to 58% [80]. In aquatic crustaceans other than ostracods, such as crabs and shrimps, repeats and TEs can make up between 50.4% and 57% [81-83] of the genome, although TE content from 7.4% to 12.9% was reported for different assemblies of *Daphnia pulex* [17,70,71]. In other, probably younger, asexuals, TE content at the genome-wide scale can exceed 50%, as for example in *Meloidogyne* root-knot nematodes [72].

4.3. TE insertion sites

One of the possible explanations for the relatively high TE content of *D. stevensoni* in our sequence data could be their genomic locations. Through partial selection of fosmids containing transposons, we might have sequenced some TE islands in the genome of *D. stevensoni* outside of coding regions. Our results detecting up to 19.4% exons in individual fosmids but only an average overlap of up to 0.01% between exons and TE and up to 2.1% between introns and TEs in the boxplot (Figure 4) support this point of view; and are illustrated for 38 selected fosmids in Figure 5a-f. These observed patterns would largely be consistent with predictions [79] on the predominantly neutral character for the bulk of TE insertions but will require genome-wide data for confirmation. The available Illumina draft genome of *D. stevensoni* is too fragmented [45] to test these assumptions further.

Deleted: 76
Deleted: 77
Deleted: 78
Deleted: data
Deleted: probably
Deleted: comparable to the
Deleted: , given that only 13 out of 104 fosmids were selected with TE-specific probes (Table S2).
Deleted: 19.1
Deleted: %
Deleted: 79
Deleted: 80
Deleted: 82
Deleted: 69
Deleted: 70
Deleted: 71
Deleted: (biased)
Deleted:
Deleted: also
Deleted: (Figure 4 and
Deleted:)
Deleted: 78
Deleted: ,
Deleted: .
Formatted: Font: Italic
Formatted: Font:

Both, the draft genome of *D. stevensoni* as well as sequenced fosmids seem to harbor recently active transposons, as supported by the estimated low substitution levels indicating recent activity (Figure 3) and from overlapping TE-rich regions of fosmid contigs (see examples in Figure 5a-f). These indications for relatively recent transposition, together with the low number of insertions within exons (Figure 4), at least partly fit the dichotomous pattern that Muszewska *et al*. described from fungal transposons [84], where young TEs were not located inside genes, while older TEs were found both inside and outside of coding regions. It is also possible that the sequenced TE islands of *D. stevensoni* in the fosmid dataset could come from pericentromeric regions, given that we did not sequence any fosmids containing telomeres. Other studies on humans [85] and rice [86], for example, found a higher abundance of TEs near centromeres. The lack of LINE-like TEs being associated with telomeres in *D. stevensoni* in fosmid sequence data also indicates that there has been no co-option of the investigated TEs for telomeric functions, as in *Drosophila* [87] or bdelloid rotifers [88].

Deleted: The genomic regions of
Deleted: here
Deleted: 83
Deleted: our
Deleted: 84
Deleted: 85
Formatted: Font: Not Italic
Deleted: 86
Deleted: 87

4.4. Assessing the impact of putatively ancient asexual reproduction on TE landscapes in non-marine ostracods

Following one of the predictions outlined in the introduction, long-term asexuality is expected to increase the transposable element load because of reduced efficiency of selection for the removal of deleterious mutations and transposons in finite populations [4,5,6]. Our results are consistent with this hypothesis. It is possible that asexual darwinulid ostracods inherited relatively high transposable loads from their sexual ancestors when abandoning sexual reproduction, which could go back as far as 200 myr ago [37] and to 25 Mya in *D. stevensoni* [38]. Glémin *et al*. implied that it might take a long time for asexuals to purge TEs from their genomes [25]. Our results indicate that even millions of years have not been sufficient for some Darwinulidae to efficiently purge their TE.

Unrestrained TE proliferation should eventually drive asexual lineages to extinction, unless asexual hosts can tightly control TE copy numbers [10-12], with putative ancient asexual bdelloids as the most striking example [10,13,14]. Eukaryotic hosts have developed a wide arsenal of molecular defense mechanisms against TEs [29,89], including for example siRNAs and piRNAs [90,91], and chromatin-based pathways [92]. The relatively high TE content and diversity in *D. stevensoni* could imply that some of these defense mechanisms are lacking or are less efficient, leading to the accumulation of transposons when these are not removed [25]. This explanation seems more likely than the lack of time for purging TEs from sexual ancestors. Alternatively, the insertion pattern of TEs in *D. stevensoni,* as suggested by our preliminary observations, may be biased towards non-coding regions, and could therefore have less deleterious effects on the host. The observed lack of insertions into exons as observed in the fosmid data is most likely explained by selection against genic insertions. Finally, certain TEs, such as *Dong*, display insertion specificity that directs them away from coding regions.

Deleted: 88
Deleted: 89
Deleted: 90
Deleted: 91

Another factor that can potentially influence transposon abundance is the presence of bacterial endosymbionts. Kraaijeveld et al. suggested that the higher copy numbers of *gypsy* transposons in asexual wasps as compared to sexual relatives [23] could partly be due to the feminizing endosymbiont *Wolbachia,* which is only present in asexuals. The *Cardinium* endosymbionts, which could have similar effects on their hosts as *Wolbachia*, have recently been described in non-marine ostracods including *D. stevensoni* [93], but their potential effect on transposable element loads in asexual ostracods remains to be investigated.

Our results based on an Illumina draft genome [45] and parts of a fosmid library indicate that asexual reproduction in the ostracod *D. stevensoni* did not substantially reduce its transposable load, as may have been the case in other asexual taxa [3,10,11,13,15,16,73]. On the contrary, it rather seems that this ostracod species is another example of an asexual being less efficient in removing TEs. Whether this is owing to the loss or dysfunction of molecular defense mechanisms controlling TE abundance, the absence of methylation, preferred insertion outside of coding regions, or other factors, remains to be investigated in future studies when comparative genomic and transcriptomic data of high quality for this and other ostracod species become available. The absence of sex in darwinulid ostracods has been estimated from fossil data to be as old as 25 myr (*D. stevensoni* [38]) up to 200 myr (the entire post-Palaeozoic family Darwinulidae, using *Alicenula* as proxy [37]), and many asexual darwinulids became extinct after the Permian-Triassic [94]. The fact that about 30 putative ancient asexual darwinulid species are still present today suggests that TE proliferation has not driven all of these species to extinction, as might be expected [11,12]. Comparisons with TE abundance in sexual ostracods and younger asexual species are required to test whether the observed patterns of TE abundance and diversity are general features related to the old age of Ostracoda of more than 400 myr [95] or to ancient asexuality of the Darwinulidae.

Supplementary Materials: The following are available online at www.mdpi.com/xxx/s1, Figure S1: Box plot of size distribution of analysed contigs from the fosmid library. Size of sequenced contigs. Figure S2: Comparison of sequence features between three groups of fosmids. Table S1: Probes used for hybridization and detection of fosmids containing TE or telomeres. Table S2: Hybridization signal of selected fosmids for sequencing. Table S3A: Contig sequence features. Table S3B: Fosmid sequence features. Table S4: Overview of possible overlap between contig ends. Table S5: Results of TE identification with Censor.

Author Contributions: Conceptualization, I.S. and I.A.; methodology, F.R. and I.A.; software, F.R.; validation, I.S., F.R., M.D. and I.A.; formal analysis, I.S., F.R., M.D. and I.A.; investigation, I.S., K.M. and I.A.; resources, I.S. and K.M..; data curation, I.S., I.A. and F.R.; writing—original draft preparation, I.S.; writing—review and editing, I.S., F.R., K.M., M.S. and I.A..; visualization, I.S., F.R., M.S. and I.A.; supervision, F.R. and I.A.; project administration, I.S.; funding acquisition, I.S., K.M. All authors have read and agreed to the published version of the manuscript.

Funding: This research was funded by Belgian Federal Science Policy (Belspo), grant number WI/36/I03. The stay of IS at Woods Hole was funded by the L. & A. Colwin Fund of the Marine Biology Laboratory, MA. MD was supported by the Blue Economy Internship Program (BEIP) of the Commonwealth of Massachusetts at the MBL.

Acknowledgments: Irina Yushenova (Woods Hole, USA) is acknowledged for helpful suggestions with the analyses, Andy Vierstraten (Gent, Belgium) and Arnaud Bellec (CNRGV, INRA, Toulouse, France) and his colleagues for technical support with fosmid sequencing, Marie Cours (Brussels, Belgium) for assistance in

Deleted: 92

Deleted: Although o

Deleted: data

Deleted: do not extend to a genome-wide scale,

Deleted: they

Deleted: 72

Deleted:

Deleted: 93

Deleted: 94

drawing the box plots, and Jeroen Venderickx (Brussels, Belgium) for providing the photograph of the *D. stevensoni* sample.

Conflicts of Interest: The authors declare no conflict of interest. The funders had no role in the design of the study; in the collection, analyses, or interpretation of data; in the writing of the manuscript, or in the decision to publish the results.

References

1. Hickey, D.A. Selfish DNA: a sexually-transmitted nuclear parasite. *Genetics*, **1982**, *101*, 519–531.
2. Zeyl, C., Bell, G., Green, D.M. Sex and the spread of retrotransposon Ty3 in experimental populations of *Saccharomyces cerevisiae*. *Genetics*, **1996**, *143*, 1567–1577.
3. Bast, J., Jaron, K.S., Schuseil, D., Roze, D., Schwander, T. Asexual reproduction reduces transposable element load in experimental yeast populations. *eLife*, **2019**, *8*, e48548. doi:10.7554/eLife.48548
4. Kondrashov, A.S. Deleterious mutations and the evolution of sexual reproduction. *Nature*, **1988**, *336*, 435–440.
5. Muller, H.J. The relation of recombination to mutational advance. *Mutat. Res*.,**1964**, *1*, 2–9.
6. Dolgin, E.S., Charlesworth, B. The fate of transposable elements in asexual populations. *Genetics*, **2006**, *174*, 817-827. 10.1534/genetics.106.060434
7. Bachtrog, D. Y-chromosome evolution: emerging insights into processes of Y-chromosome degeneration. *Nat. Rev. Genet*., **2013**, *14*, 113–124. https://doi.org/10.1038/nrg3366
8. Leung, W., Shaffer, C.D., Reed, L.K., Smith, S.T., Barshop, W., Dirkes, W., Dothager, M., Lee, P., Wong, J., Xiong, D., et al. *Drosophila* Muller F elements maintain a distinct set of genomic properties over 40 million years of evolution. *G3*, **2015**, *5*, 719–7440. 10.1534/g3.114.015966
9. Lynch, M., Blanchard, J.L. Deleterious mutation accumulation in organelle genomes. *Genetica*, **1998**, *102–103*, 29–39. https://doi.org/10.1023/A: 1017022522486
10. Arkhipova, I., Meselson, M. Transposable elements in sexual and ancient asexual taxa. *Proc. Natl. Acad. Sci. U.S.A*., **2000**, *97*, 14473-14477. https://doi.org/10.1073/pnas.97.26.14473
11. Arkhipova, I., Meselson, M. Deleterious transposable elements and the extinction of asexuals. *Bioessays*, **2005**, *27*, 76–85. 10.1002/bies.20159
12. Nuzhdin, S.V., Petrov, D.A. Transposable elements in clonal lineages: lethal hangover from sex. *Biol. J. Linn. Soc*., **2003**, *79*, 33–41.
13. Flot, J.F., Hespeels, B., Li, X., Noel, B., Arkhipova, I., Danchin, E.G., Hejnol, A., Henrissat, B., Koszul, R., Aury, J.M., et al. Genomic evidence for ameiotic evolution in the bdelloid rotifer *Adineta vaga*. *Nature*, **2013**, *500*, 453–457. https://doi.org/10.1038/nature12326
14. Arkhipova, I.R., Meselson, M. Diverse DNA transposons in rotifers of the class Bdelloidea. *Proc. Natl. Acad. Sci. U.S.A*., **2005**, *102*, 11781-11786. https://doi.org/10.1073/pnas.0505333102
15. Schaack, S., Pritham, E.J., Wolf, A., Lynch, M. DNA transposon dynamics in populations of *Daphnia pulex* with and without sex. *Proc. R. Soc. B*., **2010**, *277*, 2381–2387. https://doi.org/10.1098/rspb.2009.2253
16. Rho, M., Schaack, S., Gao, X., Kim, S., Lynch, M., Tang, H. LTR retroelements in the genome of *Daphnia pulex*. *BMC Genomics*, **2010**, *11*, 425. 10.1186/1471-2164-11-425
17. Jiang, X., Tang, H., Ye, Z., Lynch, M. Insertion polymorphisms of mobile genetic elements in sexual and asexual populations of *Daphnia pulex*. *Genome Biol. Evol*., **2017**, 9, 362–374. https://doi.org/10.1093/gbe/evw302
18. Bast, J., Schaefer, I., Schwander, T., Maraun, M., Scheu, S., Kraaijeveld, K. No accumulation of transposable elements in asexual arthropods. *Mol. Biol. Evol*., **2016**, *33*, 697-706. doi: 10.1093/molbev/msv261
19. Szitenberg, A., Cha, S., Opperman, C.H., Bird, D.M., Blaxter, M.L., Lunt, D.H. Genetic drift, not life history or RNAi, determine long-term evolution of transposable elements. *Genome Biol. Evol*., **2016**, *8*, 2964-2978. 10.1093/gbe/evw208
20. Agren, J.A., Greiner, S., Johnson, M.T., Wright, S.I. No evidence that sex and transposable elements drive genome size variation in evening primroses. *Evolution*, **2015**, *69*, 1053–1062. https://doi.org/10.1111/evo.12627
21. Zeyl, C., Bell, G., Dasilva, J. Transposon abundance in sexual and asexual populations of *Chlamydomonas reinhardtii*. *Evolution*, **1994**, *48*, 1406-1409. 10.2307/2410398
22. Abad, P., Gouzy, J., Aury, J.-M., Castagnone-Sereno, P., Danchin, E.G.J., Deleury, E., Perfus-Barbeoch, L., Anthouard, V., Artiguenave, F., Blok, V.C. et al. Genome sequence of the metazoan

plant-parasitic nematode *Meloidogyne incognita*. *Nat. Biotechnol.*, **2008**, *26*, 909–915. https://doi.org/10.1038/nbt. Abad, P., Gouzy, J., Aury, J.-M., Castagnone-Sereno, P., Danchin, E.G.J., Deleury, E., Perfus-Barbeoch, L., Anthouard, V., Artiguenave, F., Blok, V.C. et al. Genome sequence of the metazoan plant-parasitic nematode *Meloidogyne incognita*. *Nat. Biotechnol.*, **2008**, *26*, 909–915. https://doi.org/10.1038/nbt.1482

23. Kraaijeveld, K., Zwanenburg, B., Hubert, B., Vieira, C., De Pater, S., Van Alphen, J.J.M., Den Dunnen, J.T., De Knijff, P. Transposon proliferation in an asexual parasitoid. *Mol. Ecol.*, **2012**, *21*, 3898–3906. 10.1111/j.1365-294X.2012.5582
24. Simonti, C.N., Pavlicev, M., Capra, J.A. Transposable element exaptation into regulatory regions is rare, influenced by evolutionary age, and subject to pleiotropic constraints. *Mol. Biol. Evol.*, **2017**, *34*, 2856-2869. doi:10.1093/molbev/msx219
25. Glémin S, François CM, Galtier N. Genome Evolution in Outcrossing vs. Selfing vs. Asexual Species. *Methods Mol. Biol.*, **2019**, *1910*, 331-369. doi: 10.1007/978-1-4939-9074-0_11
26. Charlesworth, B., Langley, C. The evolution of self-regulated transposition of transposable elements. *Genetics*, **1986**, *112*, 359–383.
27. Startek, M., Le Rouzic, A., Capy, P., Grzebelu, D., Gambin, A. Genomic parasites or symbionts? Modeling the effects of environmental pressure on transposition activity in asexual populations. *Theor. Popul. Biol.*, **2013**, *90*, 145–151. https://doi.org/10.1016/j.tpb.2013.07.004
28. Arkhipova, I.R. Using bioinformatic and phylogenetic approaches to classify transposable elements and understand their complex evolutionary histories. *Mobile DNA,* **2017**, *8*, 19. doi:10.1186/s13100-017-0103-2
29. Bourque, G., Burns, K.H., Gehring, M., Gorbunova, V., Seluanov, A., Hammell, M., Imbeault, M., Izsvák, Z., Levin, H.L., Macfarlan, T.S., Mager, D.L., Feschotte. C. Ten things you should know about transposable elements. *Genome Biol.*, **2018**, *19*, 199. doi: 10.1186/s13059-018-1577-z
30. Huang, C.R., Burns, K.H., Boeke, J.D. Active transposition in genomes. *Ann. Rev. Gen.*, **2012**, *46*, 651–675. doi:10.1146/annurev-genet-110711-155616
31. Peccoud, J., Loiseau, V., Cordaux, R., Gilbert, C. Horizontal transfer of transposons in insects. *Proc. Natl. Acad. Sci. U.S.A*, **2017**, *114*, 4721-4726. 10.1073/pnas.1621178114
32. Schön, I., Rossetti, G., Martens, K. Ancient asexual darwinulids : ancient scandals or scandalous gossip? In *Lost sex. The evolutionary biology of parthenogenesis;* Schön, I., Martens, K., Van Dijk, Eds.; Springer Academic Publishers, Dordrecht, The Netherlands, 2009, pp. 217-240.
33. Heethoff, M., Norton, R.A., Scheu, S., Maraun, M. Parthenogenesis in oribatid mites (Acari, Oribatida): evolution without sex In *Lost sex. The evolutionary biology of parthenogenesis;* Schön, I., Martens, K., Van Dijk, Eds.; Springer Academic Publishers, Dordrecht, The Netherlands, 2009, pp. 241-258.
34. Schwander, T. Evolution: The end of an ancient asexual scandal. *Curr. Biol.*, **2016**, *26*, R233-235. 10.1016/j.cub.2016.01.034
35. Schwander, T., Lee, H., Crespi, B.J. Molecular evidence for ancient asexuality in *Timema* stick insects. *Curr. Biol.*, **2011**, *21*, 1129–1134. 10.1016/j.cub.2011.05.026
36. Mark Welch, D., Ricci, C., Meselson, M. Bdelloid rotifers: progress in understanding the success of an evolutionary scandal. In *Lost sex. The evolutionary biology of parthenogenesis;* Schön, I., Martens, K., Van Dijk, Eds.; Springer Academic Publishers, Dordrecht, The Netherlands, 2009, pp. 259-280.
37. Martens, K., Rossetti, G., Horne, D. How ancient are ancient asexuals? *Proc. R. Soc. B*, **2003**, *270*, 723–729. https://doi.org/10.1098/rspb.2002.2270
38. Straub, E.B. Mikropaläontologische Untersuchungen im Tertiär zwischen Ehingen und Ulm a.d. Donau. *Geol, Jb.*, **1952**, *66*, 433-523.
39. Schön, I., Arkhipova, I.R. Two families of non-LTR retrotransposons, *Syrinx* and *Daphne*, from the Darwinulid ostracod, *Darwinula stevensoni*. *Gene*, **2006**, *371*, 296–307.
40. Shribak, M. Polychromatic polarization microscope: bringing colors to a colorless world. *Sci. Rep.*, **2015**, *5*, 17340. https://doi.org/10.1038/srep17340

41. Tétart, J. Les garnitures chromosomiques des Ostracodes d'eau douce. *Trav. Lab. Hydrobiol. Univ. Grenoble*, **1978**, *69-70*, 113-140.
42. Frydrychová, R., Grossmann, P., Trubac, P., Vítková, M., & Marec, F.E. Phylogenetic distribution of TTAGG telomeric repeats in insects. *Genome* **2004**, *47*, 163-178. 10.1139/g03-100
43. Schön, I, Martens, K. No slave to sex. *Proc. R. Soc. Lond. B* **2003**, *270*, 827-833. https://doi.org/10.1098/rspb.2002.2314
44. Altschul, S.F., Gish, W., Miller, W., Myers, E.W. & Lipman, D.J. Basic local alignment search tool. *J. Mol. Biol.*, **1990**, *215*, 403-410. 10.1016/S0022-2836(05)80360-2
45. Tran Van, P., Anselmetti, Y., Bast, J., Dumas, Z., Galtier, N., Jaron, K., Martens, K., Parker, D., Robinson-Rechavi, M., Schwander, T., Simion, P. & Schön, I. First annotated genomes of draft genomes of three non-marine ostracods (Ostracoda, Crustacea) with different reproductive modes. *G3, accepted*.
bioRxiv 2020.12.02.409169; doi: https://doi.org/10.1101/2020.12.02.409169
46. Flutre, T., Duprat, E., Feuillet, C., Quesneville, H. Considering transposable element diversification in *de novo* annotation approaches. *PLoS One*, **2011**, *6*, e16526. doi: 10.1371/journal.pone.0016526
47. Quesneville, H., Bergman, C.M., Andrieu, O., Autard, D., Nouaud, D., Ashburner, M., Anxolabehere, D. Combined evidence annotation of transposable elements in genome sequences. *PLoS Computat. Biol.*, **2005**, *1*, 166–175. doi:10.1371/journal.pcbi.0010022
48. Wicker, T., Sabot, F., Hua-Van, A., Bennetzen, J.L., Capy, P., Chalhoub, B., Flavell, A., Leroy, P., Morgante, M., Panaud, O., et al. A unified classification system for eukaryotic transposable elements. *Nat. Rev. Genet.*, **2007**, *8*, 973–982. https://doi.org/10.1038/nrg2165
49. Smit, A.F.A., Hubley, R., Green, P. RepeatMasker Open-4.0. 2013-2015. Available online: http://www.repeatmasker.org.
50. Stanke, M., Schöffmann, O., Morgenstern, B., Waack, S. Gene prediction in eukaryotes with a generalized hidden Markov model that uses hints from external sources. *BMC Bioinform.*, **2006**, *7*, 62. https://doi.org/10.1186/1471-2105-7-62
51. Quinlan, A.R., Hall, I.M. BEDTools: a flexible suite of utilities for comparing genomic features. *Bioinformatics*, **2010**, *26*, 841-842. doi:10.1093/bioinformatics/btq033
52. Wickham, H. *ggplot2: Elegant Graphics for Data Analysis*. 2016. Springer-Verlag New York.
53. R Core Team. R: A language and environment for statistical computing. 2013. R Foundation for Statistical Computing, Vienna, Austria. http://www.R-project.org/.Author 1, A.B. (University, City, State, Country); Author 2, C. (Institute, City, State, Country). Personal communication, 2012.
54. Krzywinski, M., Schein, J., Birol, I., Connors, J., Gascoyne, R., Horsman, D., Jones, S.J., Marra, M.A. Circos: an information aesthetic for comparative genomics. *Genome Res.*, **2009**. 19, 1639-1645. 10.1101/gr.092759.109
55. Waterhouse, R.M., Seppey, M., Simão, F.A., Manni, M., Ioannidis, P., Klioutchnikov, G., Kriventseva, E.V., Zdobnov, E.M. BUSCO Applications from Quality Assessments to Gene Prediction and Phylogenomics, *Mol. Biol. Evol.*, **2018**, *35*, 543-548. https://doi.org/10.1093/molbev/msx319
56. Simão, F.A., Waterhouse, R.M., Ioannidis, P., Kriventseva, E.V., Zdobnov, E.M. BUSCO: assessing genome assembly and annotation completeness with single-copy orthologs. *Bioinformatics*, **2015**, *31*, 3210-3212. https://doi.org/10.1093/bioinformatics/btv351
57. Edgar RC: MUSCLE: a multiple sequence alignment method with reduced time and space complexity. *BMC Bioinformatics* 2004, 5:113.
58. Zimmermann, L., Stephens, A., Nam, S.Z., Rau, D., Kubler, J., Lozajic, M., Gabler, F., Soding, J., Lupas, A.N., Alva, V. A completely reimplemented MPI bioinformatics toolkit with a new HHpred server at its core. *J. Mol. Biol.*, **2018**, 430, 2237-2243. 10.1016/j.jmb.2017.12.007
59. Waterhouse, A.M., Procter, J.B., Martin, D.M.A., Clamp, M., Barton, G.J. Jalview Version 2—a multiple sequence alignment editor and analysis workbench. *Bioinformatics*, **2009**, *25*, 1189-1191. 10.1093/bioinformatics/btp033

Deleted: ¶
Formatted: Font: 10 pt, English (US)
Formatted: Font: 10 pt, English (US)
Formatted: Font: 10 pt, English (US)
Formatted: Font: 10 pt, English (US)
Formatted: Left, Indent: Left: 0 cm, Hanging: 0,75 cm
Formatted: Font: 10 pt, English (US)
Formatted: Font: 10 pt, English (US)
Formatted: Font: 10 pt, English (US)
Formatted: Font: 10 pt, English (US)
Formatted: Font: 10 pt, English (US)
Formatted: Font: 10 pt, English (US)
Formatted: Font: 10 pt, English (US)
Formatted: Font: 10 pt, English (US)
Formatted: Font: 10 pt, English (US)
Formatted: Font: 10 pt, Italic
Formatted: Font: 10 pt
Formatted: Font: 10 pt

60. Burke, W.D., Malik, H.S., Jones, J.P., Eickbush, T.H. The domain structure and retrotransposition mechanism of R2 elements are conserved throughout arthropods. *Mol. Biol. Evol.,* **1999**, *16,* 502-511. 10.1093/oxfordjournals.molbev.a026132
61. Volff, J.N., Körting, C., Froschauer, A., Sweeney, K., Schartl, M. Non-LTR retrotransposons encoding a restriction enzyme-like endonuclease in vertebrates. *J. Mol. Evol.,* **2001**, *52*, 351-360. 10.1007/s002390010165
62. Biedler, J.K., Chen, X., Tu, Z. Horizontal transmission of an R4 clade non-long terminal repeat retrotransposon between the divergent *Aedes* and *Anopheles* mosquito genera. *Insect Mol. Biol.,* **2015**, *24*, 331-337. 10.1111/imb.12160
63. Shivram, H., Cawley, D., Christensen, S.M. Targeting novel sites: The N-terminal DNA binding domain of non-LTR retrotransposons is an adaptable module that is implicated in changing site specificities. *Mob. Genet. Elements,* **2011**, 1, 169-178. 10.4161/mge.1.3.18453
64. Kojima, K.K., Fujiwara, H. Cross-genome screening of novel sequence-specific non-LTR retrotransposons: various multicopy RNA genes and microsatellites are selected as targets. *Mol. Biol. Evol.,* **2004**, 21, 207-217. 10.1093/molbev/msg235
65. Deng, Y., Liu, J., Zheng, Q., Yong, W., Lu, M. Structures and polymorphic interactions of two heptad-repeat regions of the SARS virus S2 protein. *Structure,* **2006**, *14*, 889-899. 10.1016/j.str.2006.03.007
66. Cai, Y., Zhang, .J, Xiao, T., Peng, H., Sterling, S.M., Walsh, R.M., Jr., Rawson, S., Rits-Volloch, S., Chen, B. Distinct conformational states of SARS-CoV-2 spike protein. *Science,* **2020**, *369*, 1586-1592. 10.1126/science.abd4251
67. Dietrich, M.H., Ogden, K.M., Long, J.M., Ebenhoch, R., Thor, A., Dermody, T.S., Stehle, T. Structural and functional features of the reovirus σ1 tail. *J. Virol.,* **2018**, *92*, 14. 10.1128/JVI.00336-18
68. Malik, H.S., Henikoff, S., Eickbush, T.H. Poised for contagion: evolutionary origins of the infectious abilities of invertebrate retroviruses. *Genome Res.,* **2000**, *10*, 1307-1318. 10.1101/gr.145000
69. Rodriguez, F., Kenefick, A.W., Arkhipova, I.R. LTR-retrotransposons from bdelloid rotifers capture additional ORFs shared between highly diverse retroelement types. *Viruses,* **2017**, *9*, 78. 10.3390/v9040078
70. Colbourne, J.K., Pfrender, M.E., Gilbert, D., Thomas, W.K., Tucker, A., Oakley, T.H., Tokishita, S., Aerts, A., Arnold, G.J., Basu, M.K., et al. The ecoresponsive genome of *Daphnia pulex*. *Science*, **2011**, *331*, 555–561. 10.1126/science.1197761
71. Ye, Z., Xu, S., Spitze, K., Asselman, J., Jiang, X., Ackerman, M.S., Lopez, J., Harker, B., Raborn, R.T., Thomas, W.K., Ramsdell, J. A new reference genome assembly for the microcrustacean *Daphnia pulex*. *G3: Genes, Genomes, Genetics,* **2017**, *7*, 1405-1416. https://doi.org/10.1534/g3.116.038638
72. Blanc-Mathieu, R., Perfus-Barbeoch, L., Aury, J.M., Da Rocha, M., Gouzy, J., Sallet, E., Martin-Jimenez, C., Bailly-Bechet, M., Castagnone-Sereno, P., Flot, J.F. et al. Hybridization and polyploidy enable genomic plasticity without sex in the most devastating plant-parasitic nematodes. *PLoS Genet*., **2017**, *13*, e1006777. https://doi.org/10.1371/journal.pgen.1006777
73. Schaack, S., Gilbert, C., Feschotte, C. Promiscuous DNA: horizontal transfer of transposable elements and why it matters for eukaryotic evolution. *Trends Ecol. Evol.*, **2010**, *25*, 537–546. doi:10.1016/j.tree.2010.06.001
74. Dupeyron, M., Leclercq, S., Cerveau, N., Bouchon, D., Gilbert, C. Horizontal transfer of transposons between and within crustaceans and insects. *Mobile DNA*, **2014**, *5*, 4. doi:10.1186/1759-8753-5-4
75. Wallau, G.L., Vieira, C., Loreto, É. Genetic exchange in eukaryotes through horizontal transfer: connected by the mobilome. *Mobile DNA*, **2018**, *9*, 6. doi:10.1186/s13100-018-0112-9
76. Metzger, M.J., Paynter, A.N., Siddall, M.E., Goff, S.P. Horizontal transfer of retrotransposons between bivalves and other aquatic species of multiple phyla. *Proc. Natl. Acad. Sci. U.S.A.*, **2018**, *115*, E4227-E4235. doi:10.1073/pnas.1717227115

77. Arkhipova, I.R., Rodriguez, F. Genetic and epigenetic changes involving (retro)transposons in animal hybrids and polyploids. *Cytogenet*. *Genome Res*., **2013**, *140*, 295-311. https://doi.org/10.1159/000352069
78. Huang, C.R., Burns, K.H., Boeke, J.D. Active transposition in genomes. *Ann. Rev. Gen*., **2012**, *46*, 651–675. doi:10.1146/annurev-genet-110711-155616
79. Arkhipova, I.R. Neutral theory, transposable elements, and eukaryotic genome evolution. *Mol. Biol. Evol*., **2018**, *35*, 332-1337. doi: 10.1093/molbev/msy083
80. Petersen, M., Armisén, D., Gibbs, R.A., Hering, L., Khila, A., Mayer, G., Richards, S., Niehuis, O., Misof, B. Diversity and evolution of the transposable element repertoire in arthropods with particular reference to insects. *BMC Evol. Biol*., **2019**, *19*, 11. doi: 10.1186/s12862-018-1324-9
81. Song, L., Bian, C., Luo, Y., Bian, C., Luo, Y., Wang, L., You, X., Li, J., *et al*. Draft genome of the Chinese mitten crab, *Eriocheir sinensis*. *GigaSci*, **2016**, *5*, 5. doi:10.1186/s13742-016-0112-y
82. Yuan, J., Gao, Y., Zhang, X., Wei, J., Liu, C., Li, F., Xiang, J. Genome sequences of marine shrimp *Exopalaemon carinicauda* Holthuis provide insights into genome size evolution of Caridea. *Marine drugs*, **2017**, *15*, 213. doi:10.3390/md15070213
83. Kao, D., Lai, A.G., Stamataki, E., Rosic, S., Konstantinides, N., Jarvis, E., Di Donfrancesco, A., Pouchkina-Stancheva, N., Semon, M., Grillo, M., Bruce, H. The genome of the crustacean *Parhyale hawaiensis*, a model for animal development, regeneration, immunity and lignocellulose digestion. *Elife*, **2016**, *5*, e20062. 10.7554/eLife.20062
84. Muszewska, A., Steczkiewicz, K., Stepniewska-Dziubinska, M. et al. Transposable elements contribute to fungal genes and impact fungal lifestyle. *Sci. Rep.*, **2019**, 9, 4307. doi:10.1038/s41598-019-40965-0
85. Hartley, G., O'Neill, R.J. Centromere repeats: hidden gems of the genome. *Genes*, **2019**, *10*, 223. doi:10.3390/genes10030223
86. Gao, D., Jiang, N., Wing, R.A., Jiang, J., Jackson, S.A. Transposons play an important role in the evolution and diversification of centromeres among closely related species. *Front. Plant Sci.*, **2015**, *6*, 216. doi:10.3389/fpls.2015.00216
87. Pardue, M.-L., DeBaryshe, P.G. Retrotransposons that maintain chromosome ends. *Proc. Natl. Acad. Sci. U.S.A*., **2011**, *108*, 20317–20324. https://doi.org/10.1073/pnas.1100278108
88. Gladyshev, E.A., Arkhipova, I.R. Telomere-associated endonuclease-deficient Penelope-like retroelements in diverse eukaryotes. *Proc. Natl. Acad. Sci. U.S.A*., **2007**, *104*, 9352-9357. https://doi.org/10.1073/pnas.0702741104
89. Goodier, J.L. Restricting retrotransposons: a review. *Mobile DNA*, **2016**, *7*, 16. doi:10.1186/s13100-016-0070-z
90. Rodriguez, F., Arkhipova, I.R. Multitasking of the piRNA silencing machinery: targeting transposable elements and foreign genes in the bdelloid rotifer *Adineta vaga*. *Genetics*, **2016**, *203*, 255-268. doi: 10.1534/genetics.116.186734
91. Rodriguez, F., Arkhipova, I.R. Multitasking of the piRNA silencing machinery: targeting transposable elements and foreign genes in the bdelloid rotifer *Adineta vaga*. *Genetics*, **2016**, *203*, 255-268. doi: 10.1534/genetics.116.186734
92. Molaro, A., Malik, H.S. Hide and seek: how chromatin-based pathways silence retroelements in the mammalian germline. *Curr. Opin. Genet. Dev*., **2016**, *37*, 51–58. doi: 10.1016/j.gde.2015.12.001
93. Schön, I., Kamiya, T., Van den Berghe, T., Van den Broecke, L., Martens, K. The evolutionary history of novel *Cardinium* strains and their non-marine ostracod (Crustacea) hosts. *Mol. Phyl. Evol.*,**2019**,*130*, 406-415. 10.1016/j.ympev.2018.09.008
94. Martens, K., Horne, D.J., Griffiths, H.I. Age and diversity of non-marine ostracods. In: *Sex and Parthenogenesis. Evolutionary Ecology of Reproductive Modes in Non-Marine Ostracods*; Martens, K., Ed.; Backhuys Publishers, Leiden, 1998, pp. 37–55.
95. Wolfe, J.M., Daley, A.C., Legg, D.A., Edgecombe, G.D. Fossil calibrations for the arthropod Tree of Life. *Earth-Sci. Rev*., **2016**, *160*, 43-110. https://doi.org/10.1016/j.earscirev.2016.06.008

Publisher's Note: MDPI stays neutral with regard to jurisdictional claims in published maps and institutional affiliations.

© 2020 by the authors. Submitted for possible open access publication under the terms and conditions of the Creative Commons Attribution (CC BY) license (http://creativecommons.org/licenses/by/4.0/).
