# Supplementary material for: A Survey of Transposon Landscapes in the Putative Ancient Asexual Ostracod Darwinula stevensoni
Source: Genes (Basel). 2021 Mar 11;12(3):401. doi: 10.3390/genes12030401 (PMC7998251; doi:10.3390/genes12030401)
Supplement: Supplementary file 1 [file genes-12-00401-s001.zip › supplementary material_resubmission/Figure S1_box plot.docx]

**
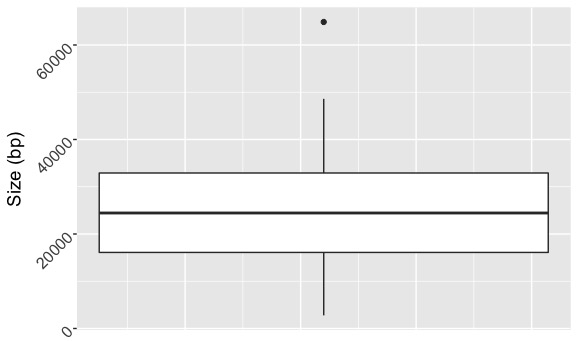
**

**Figure S1**: **Box plot of size distribution of analysed contigs from the fosmid library.** The size range of fosmids is provided in basepairs (bp). Boxes contain the interquartile range from the 25^th^ to the 75^th^ percentile, the horizontal line indicates the median, and vertical lines minimum and maximum distributions of the data. Outliers are shown by dots.
