# Supplementary material for: A Survey of Transposon Landscapes in the Putative Ancient Asexual Ostracod Darwinula stevensoni
Source: Genes (Basel). 2021 Mar 11;12(3):401. doi: 10.3390/genes12030401 (PMC7998251; doi:10.3390/genes12030401)
Supplement: Supplementary file 1 [file genes-12-00401-s001.zip › supplementary material_resubmission/Table S1_hybridisation probes.docx]

**Table S1: Overview of probes used for hybridization of the fosmid library of *Darwinula stevensoni*.**

| **Direction** | **Target** | **Sequence** |
| --- | --- | --- |
| Sense | L1-like Syrinx | TCCTATCTGACCGAGAGATCGTTC |
| Antisense | L1-like Syrinx | CTTACCCTGAACCCCTGAACGATC |
| Sense | LINE-like | ATGGATAGGATCTGTAGGCGCAGC |
| Antisense | LINE-like | GACGTCACCGATATTGACTCC |
| Sense | Marine-like | TCA GAA GTT GGC TGG CGT CAT TCA |
| Antisense | Marine-like | TTT GAG ACG CTC TTT TTG AAT GAC |
| Sense | Telomeric repeats | GTTAGGTTAGGTTAGGTTAGGTAG |
| Antisense | Telomeric repeats | CCTAACCTAACCTAACCTACCTAA |
