# Supplementary material for: A Survey of Transposon Landscapes in the Putative Ancient Asexual Ostracod Darwinula stevensoni
Source: Genes (Basel). 2021 Mar 11;12(3):401. doi: 10.3390/genes12030401 (PMC7998251; doi:10.3390/genes12030401)
Supplement: Supplementary file 1 [file genes-12-00401-s001.zip › supplementary material_resubmission/Figure S2_TE_ms.docx]

**Figure S1:** **Comparison of sequence features between three groups of fosmids.**

We distinguish three groups: all fosmids, the majority of fosmids that had been selected with probes for TE (TE-probes) and an additional 11 fosmids that had been selected with probes for coding genes (gene probes). The figure shows average frequencies of as % of total fosmid lengths of various features. TE_RM - TE as predicted with RepeatMasker; TE_Censor – TE predicted with Censor; overlap TE_exon and overlap TE_intron were estimated with translated TE as predicted with Censor.
