# Supplementary material for: A Survey of Transposon Landscapes in the Putative Ancient Asexual Ostracod Darwinula stevensoni
Source: Genes (Basel). 2021 Mar 11;12(3):401. doi: 10.3390/genes12030401 (PMC7998251; doi:10.3390/genes12030401)
Supplement: Supplementary file 1 [file genes-12-00401-s001.zip › supplementary material_resubmission/Table S4_Overview possible overlap between contigs.docx]

**Table S4: Overview of possible overlap between contig ends.**

Ds_ctg and Ionexpress refer to Tables S4 and S5.

| **Contig**  **Ds_ctg** | **Fosmid**  **Ionexpress** | **Overlap with contigs** | **bp overlap of total length; % identity** | **Fully included in contig** | **TE included in overlap** | **Comment** |
| --- | --- | --- | --- | --- | --- | --- |
| 1 | 1 | 32, 110, 161, 170, 190, 199, 292, 304 | 6815bp, total length; 100% |  | yes | Recent transposition |
| 11 | 006_7 | 125 | 4175bp of 4175 bp total contig length; 100% |  | no | Contig 11 removed |
| 12 | 007_1 | 338, 334, 79, 336, 341 | 1184bp; 99% |  | yes | Recent transposition |
| 17 | 009_1 | 51 | 3829bp of 3833bp total contig length; 99.87% match | 51 | no | Contig 17 removed as 51 is 16479bp long |
| 19 | 009_7 | 51 | 7703bp of 7749bp total contig length; 99.86% | 51 | no | Contig 19 removed as 51 is 16479bp long |
| 27 | 012_2 | 227, 3’ | 3107bp of 8218bp total contig length; 99.87% match |  | 200bp mariner | 3107bp of contig 27 removed |
| 33 | 014_2 | 111, 131, 291, 305 | 5102bp, full length;, 99.98% |  | yes | Recent transposition |
| 39 | 016_3 | 52 | 955bp of 6812bp total contig length; 100% |  | no | 955 bp of contig 39 removed |
| 41 | 016_10 | 57, 289 | 3113bp of 3113bp total contig length; 100% |  | yes | Recent transposition |
| 46 | 018_1 | 60 | 3002/3211bp; 100% |  | yes | Recent transposition |
| 48 | 018_3 | 59 | 4443bp/44443bp; 100% |  | no | Contig 48 removed |
| 52 | 020_3 | 39 | 5813/6812bp; 100% |  | no | 5813 bp of contig 52 removed |
| 54 | 021_1 | 40 | 3704/3706bp; 100% | 40 | 161bp hAT; 63bp mariner | Contig 54 removed as 40 is 10625bp long |
| 66 | 024_1 | 202 | 1920bp/3271bp total contig length; 100% |  | 89bp mariner | 1920 bp of contig 66 removed |
| 90 | 030_7 | 198 | 211/236bp; 92.42% |  |  | Very short contig, removed |
| 96 | 032_1 | 115 | 1794bp/28259  bp; 99.82% |  | yes | Recent transposition |
| 107 | 035_1 | 235, 236 | 4026bp/20998 bp total contig length; 99.08% |  | no | 4026bp of contig 107 removed |
| 109 | 035_5 | 238 | 2243bp/2281bp, total contig length; 98.71% | 238 | no | Contig109 removed as 238 is 6784bp long |
| 115 | 038_1 | 96 | 4377bp/ 6976bp total contig length; 99.91% |  | yes | Recent transposition |
| 117 | 039_2 | 187 | 4955bp/4955 bp total contig length; 100% |  | yes | Recent transposition |
| 119 | 039_4 | 189 | 4252bp/4252 bp total contig length; 100% |  | 752bp mariner | Contig 119 removed |
| 120 | 039_5 | 185 | 3292bp/3292 bp total contig length; 99.97% |  | 254bp LINE/RTEBov | Contig 120 removed |
| 129 | 042_1 | 32, 292 | 2851/2851bp total contig length; 98.8% |  | yes | Recent transposition |
| 162 | 050_5 | 195 | 2799bp/2799 bp total contig length; 100% |  | no | Contig 162 removed |
| 184 | 056_1 | 118 | 7954bp/8156bp total contig length; 100% | 118 | yes | Recent transposition |
| 186 | 056_3 | 118 | 2767bp/2945 bp total contig length; 100% | 118 | yes | Recent transposition |
| 194 | 057_7 | 171 | 2118bp/2118 bp total contig length; 99.98% |  | no | Contig 194 removed as 171 is 11565bp long |
| 197 | 057_16 | 200 | 5084bp/5084 bp total contig length; 100% |  | 802bp of EnSpm, AT, Harbinger | Contig 197 removed |
| 201 | 058_10 | 191 | 1695bp/1695bp; 100% |  | no | Contig 201 removed as 191 is 6330bp long |
| 205 | 059_4 | 162, 192 | 2102bp/2605bp total contig length; 99.81% |  | no | Contig 205 removed as 162 & 192 are longer |
| 211 | 062_1 | 225, 232 | 130bp/130 bp total length; 98.41% |  | no | Very short contig, removed |
| 216 | 062_7 | 179 | 1768bp/1768bp total contig length; 100% |  | 142bp of gypsy & CR1 | Contig 216 removed as 179 is with 38715bp much longer |
| 217 | 062_13 | 179 | 2216bp/2216bp total contig length; 100% |  | no | Contig 217 removed as 179 is with 38715bp much longer |
| 218 | 062_25 | 222 | 4969bp/5456bp total contig length; 100% |  | 1234bp mariner | Contig 218 removed |
| 219 | 062_33 | 221 | 3662bp/5184bp total contig length; 99.95%% |  | 2085bp of RTEX | Recent partial transposition |
| 220 | 062_58 | 179 | 4222bp/4230bp total contig length; 99.92% |  | 214bp mariner | Contig 220 removed as 179 is with 38715bp much longer |
| 224 | 063_15 | 179 | 3544bp/3544bp total contig length; 100% |  | 100% mariner | Recent partial transposition |
| 225 | 063_26 | 232 | 1736bp/1776bp total contig length; 98.4 & 95.4% |  | 326bp Daphne | Contig 225 removed |
| 230 | 064_6 | 28 | 1888bp/1900bp total contig length; 100% |  | 362bp MULE | Contig 230 removed |
| 249 | 070_1 | 250 | 1251bp/1889bp total contig length; 95% |  | no | Contig 249 removed |
| 259 | 072_6 | 25 | 1305bp/1389bp total contig length; 99.62% |  | Almost 100% Syrinx | Recent transposition |
| 262 | 073_3 | 272 | 2205bp/2205bp total contig length; 100% |  | no | Contig 262 removed |
| 265 | 074_2 | 124 | 2835bp/2953bp total contig length; 100% | 124 | no | Contig 265 removed as 124 is 12086bp long |
| 268 | 075_2 | 83, 247, 257 | 1202bp/1503bp bp total contig length; 95.7% |  | 100% mariner | Recent transposition |
| 283 | 079_2 | 84 | 2402bp/2402bp total contig length; 100% | 84 | no | Contig 283 removed as 84 is 24817bp long |
| 284 | 079_4 | 84 | 18968bp/19119bp total contig length; 100% |  | 725bp Merlin; 108bp gypsy | Contig 283 removed as 84 is 24817bp long |
| 286 | 080_2 | 38 | 4537bp/6825bp total contig length @ 5’ end; 99.98% |  | no | 4537bp of contig 286 removed |
| 288 | 080_7 | 39, 52 | 1155bp/1159 bp total contig length; 99.99% |  | no | Contig 288 removed as 39 is with 6812bp longer |
| 290 | 080_13 | 53, 55 | 3867bp/7151bp total contig length; 100% |  | Yes; 1414bp gypsy & mariner | 3867bp of contig 290 removed |
| 301 | 087_2 | 96 | 2212bp/3144bp total contig length; 100% | 96 | no | Contig 301 removed as 96 is with 28275bp longer |
| 302 | 087_3 | 116 | 189bp/189bp; 100% | 116 |  | Contig 302 removed |
| 303 | 087_5 | 96, 116 | 4715bp/4715bp of total contig length; 100% |  | Yes; 100% LINE/RTE BovB | Recent transposition |
| 305 | 088_3 | 111 (also matches with 33, 131, 292) | 7622bp/14361 bp total contig length; 100% |  | Yes; TC1 & MULE | Recent transposition |
| 306 | 088_4 | 32 (161, 292) | 3906bp/9103 bp total contig length; 99.36% |  | Yes; mariner & TEX (Censor) & MULE (RM) | Recent transposition |
| 323 | 094_7 | 49, 141, 247 | 2239bp/4901bp total contig length; 94.69% |  | Yes; CR1; | Recent transposition |
| 329 |  | 340 | 13688bp/25552bp total contig length; 99.26% |  | no | 13688bp of contig 329 removed |
| 330 |  | 329 | 14495bp/18809 bp of total contig length; 99.92%% | 329 | 1761bp mariner | 14495bp of contig 330 removed |
| 335 |  | 338 | 1310bp/29113 bp total contig length; 100% |  | no | 1310bp of contig 335 removed |
